# Priority planting area planning for cash crops under heavy metal pollution and climate change: A case study of *Ligusticum chuanxiong* Hort

**DOI:** 10.3389/fpls.2023.1080881

**Published:** 2023-02-01

**Authors:** Ping He, Yunfeng Li, Tongtong Huo, Fanyun Meng, Cheng Peng, Ming Bai

**Affiliations:** ^1^ Faculty of Geographical Science, Beijing Normal University, Beijing, China; ^2^ Hebei Province Key Laboratory of Research and Development of Traditional Chinese Medicine, Chengde Medical University, Chengde, China; ^3^ School of Pharmacy, Chengdu University of Traditional Chinese Medicine, Chengdu, China; ^4^ Key Laboratory of Zoological Systematics and Evolution, Institute of Zoology, Chinese Academy of Sciences, Beijing, China

**Keywords:** niche model, soil cadmium pollution, scenario simulation model, *Ligusticum chuanxiong* Hort, priority conservation area

## Abstract

**Introduction:**

Soil pollution by heavy metals and climate change pose substantial threats to the habitat suitability of cash crops. Discussing the suitability of cash crops in this context is necessary for the conservation and management of species. We developed a comprehensive evaluation system that is universally applicable to all plants stressed by heavy metal pollution.

**Methods:**

The MaxEnt model was used to simulate the spatial distribution of *Ligusticum chuanxiong* Hort within the study area (Sichuan, Shaanxi, and Chongqing) based on current and future climate conditions (RCP2.6, RCP4.5, RCP6.0, and RCP8.5 scenarios). We established the current Cd pollution status in the study area using kriging interpolation and kernel density. Additionally, the three scenarios were used in prediction models to simulate future Cd pollution conditions based on current Cd pollution data. The current and future priority planting areas for *L. chuanxiong* were determined by overlay analysis, and two levels of results were obtained.

**Results:**

The results revealed that the current first- and secondary-priority planting areas for *L. chuanxiong* were 2.06 ×10^3^ km^2^ and 1.64 ×10^4^ km^2^, respectively. Of these areas, the seven primary and twelve secondary counties for current *L. chuanxiong* cultivation should be given higher priority; these areas include Meishan, Qionglai, Pujiang, and other regions. Furthermore, all the priority zones based on the current and future scenarios were mainly concentrated on the Chengdu Plain, southeastern Sichuan and northern Chongqing. Future planning results indicated that Renshou, Pingwu, Meishan, Qionglai, Pengshan, and other regions are very important for *L. chuanxiong* planting, and a pessimistic scenario will negatively impact this potential planting. The spatial dynamics of priority areas in 2050 and 2070 clearly fluctuated under different prediction scenarios and were mainly distributed in northern Sichuan and western Chongqing.

**Discussion:**

Given these results, taking reasonable measures to replan and manage these areas is necessary. This study provides. not only a useful reference for the protection and cultivation of *L. chuanxiong*, but also a framework for analyzing other cash crops.

## 1 Introduction

Soil pollution by heavy metals is pervasive worldwide, posing a threat to agricultural, livestock and fishery productivity and further jeopardizing food safety and human health since metals are not biodegradable (Li et al., 2014; Yang et al., 2018; Li M. et al., 2020). Every year, over 6,000 tons of Cr and 1.6×10^5^ tons of Pb accumulate in the soil environment globally, resulting in serious soil metal pollution (Chen et al., 2016). In China, approximately 10 million hectares of arable land and 12 million tons of grain are polluted each year by heavy metals (Qin et al., 2021; Zhao et al., 2015). In addition, heavy metals have been continually reported as the primary metal substances polluting agricultural land in developed countries such as the United States, Britain, and Japan (Nicholson et al., 2003; Arao et al., 2010; Olawoyin et al., 2012; Cho et al., 2019). Cd is one of the five most toxic heavy metals in polluted soil and has a biological half-life of 10 to 30 years; it has been listed as the first of eight major heavy metals polluting soil (Qin et al., 2021b). In China, the Cd overlimit rate was 16.1% of the total soil, leading to the contamination of approximately 1.3×10^4^ ha of land by Cd every year (Gu and Zhou, 2002; Zou et al., 2021). The contamination of land with toxic elements poses a considerable threat to the spatial distribution of plants, especially cash crops, thereby affecting agricultural yield and economic income (Rocco et al., 2016; Zwolak et al., 2019). In many countries, especially developed countries, strict land management measures have been implemented in response to different pollution levels (WHO, 2004; Fayiga and Saha, 2016; Li et al., 2019). The Ministry of Ecology and Environment of the People’s Republic of China has clearly stipulated that no green crops should be grown on heavily polluted land, further reducing the possible planting area of cash crops (MEP and MLR, 2014; Zhao et al., 2015; Huang et al., 2019). Although the climate environmental conditions are highly suitable for cash crops, the living space and geographical distribution of the plants will shrink significantly owing to soil pollution by heavy metals (Yang and Li, 2000; Lin and Ho, 2003; Chen, 2007). Therefore, it is particularly important for cash crop cultivation to determine the zones in the suitable distribution region where the concentration of pollutants is at safe levels. Identifying these priority areas not only secures crop yields, but also protects them from the threat of soil contamination.

Additionally, the content of toxic heavy metal elements in the pedosphere varies over time, depending, on the one hand, on the self-purification ability of the soil and on the degree of heavy metal pollution on the other hand (Su, 2014; Hanfi et al., 2020). The self-purification capacity of soil and the accumulation of heavy metal elements in soil are, in effect, dynamic processes, and whether soil pollution by heavy metals will continue to decrease depends on whether this dynamic process is disrupted (Moolenaar and Lexmond, 1998; Zhang et al., 2013). The area of polluted land varies greatly over time because the anthropogenic and natural sources of pollution are different across periods (Zhang and Wang, 2020). For instance, the land use pattern in the Xiangxi River watershed fluctuated substantially over 15 years due to varying degrees of human activities in this region (Wang et al., 2018). Therefore, on a certain temporal and spatial scale, understanding how land polluted by heavy metals will change and how these changes will impact the distribution of suitable areas for cash crops is an important challenge. Given that some changes will occur in polluted areas after a few years, the question is: how should the priority suitable planting zones of cash crops be divided? The identification of priority suitable planting regions of cash crops in the future depends on the results obtained by evaluating the future habitat suitability of the species.

In our study, first, a simulation model for soil pollution by heavy metals was established to predict future pollution conditions within the study zones (Wu, 2008; He et al., 2020; Hou et al., 2022). Soil heavy metal accumulation prediction models are based on the law of mass conservation, and the accumulation and purification of heavy metal pollutants in the soil are in a dynamic equilibrium when potentially toxic elements enter the soil (He et al., 2020; Zhang J. et al., 2016). To quantify the change dynamics of soil Cd pollution, simulations based on three scenario prediction models (optimistic, default, and pessimistic, representing three states of economic development) was performed to estimate the content of soil Cd at some time in the future (Wu, 2008; Yin et al., 2018; He et al., 2020). In recent years, this modeling approach has been widely used in heavy metal risk evaluation due to its substantial advantages, such as simple operation, short run time, and uncomplicated basic data input (Wu, 2008; Xiong, 2011; Yan, 2016). Habitat suitability assessments of species in the future must consider the impact of climate change on species habitats (Zhang et al., 2020; del Río et al., 2021). In a few decades, habitat suitability will vary widely because the climate is changing at an unprecedented rate, leading to extremely unstable surrounding environmental conditions (Troia et al., 2019). The global temperature is currently rising by 0.2 ± 0.1°C every ten years, and the average temperature of Earth may increase by 0.3 to 4.5°C by 2100 (Stocker et al., 2013). Climate change has already substantially altered the spatial ranges of species, continuously altering the suitability of habitats for plants and animals (Hansen et al., 2001; Davis et al., 2005). In 80 years later, more than half of species will be threatened, and European plants may face a greater risk of extinction than organisms in other regions (Thuiller et al., 2005). To accurately evaluate the spatial distribution of species based climate change, various species distribution models (SDMs), such as the genetic algorithm for rule set production (GARP), random forest (RF), and maximum entropy (MaxEnt), have been used for decades (Townsend Peterson et al., 2007; Evans et al., 2011; Wang W. et al., 2021). Recently, MaxEnt has been widely used in a variety of disciplines because of its important advantages: ease of operation, good performance, and short run time (Phillips et al., 2017; Srivastava et al., 2018). For instance, by establishing a climate distribution model for giant pandas with four climate change scenarios, researchers found that isolated, fragmented giant panda populations will be more vulnerable to extinction risk than other populations (Kong et al., 2021). In addition, MaxEnt modeling has yielded excellent suitability distribution maps and driving factors for some significantly endangered plants and animals (Preau et et al., 2018; Li et al., 2019; He et al., 2021). Thus, we used this modeling approach to simulate the current and future spatial distributions of a critical cash crop (*Ligusticum chuanxiong* Hort), based on current and future climates.

*L. chuanxiong* is a traditional Chinese cash crop which was first recorded in the Classic of Mountains and Rivers as approximately 4000 years old, but first appeared in the Divine Husbandman’s Classic of the Materia Medica as a valuable medical plant, with the functions of promoting blood and qi circulation, expelling wind, and alleviating pain (Li et al., 2012; Yuan et al., 2020). The main production areas for *L. chuanxiong*, are centered in Sichuan Province, include Pengzhou, Dujiangyan, Pengshan, Xinjin District, and other regions; Shaanxi Province and Chongqing are also distribution zones (Zhou et al., 2014; Ning et al., 2015; Xuan et al., 2019). These regions produce approximately 90% of the world’s medicinal *L. chuanxiong* (Ren et al., 2016). It is a plant easily rich in Cd. Excessive accumulation of Cd in *L. chuanxiong* affects photosynthesis and the growth of *L. chuanxiong*, inhibiting the formation of secondary metabolites such as ferulic acid and ligustrazine (Han et al., 2008). In recent years, *L. chuanxiong* has been severely threatened by soil Cd pollution in these regions, resulting in the destruction or return of a plethora of *L. chuanxiong* that had been exported (Zeng et al., 2020; Lv et al., 2021), which is the critical reason why *L. chuanxiong* was chosen as the indicator crop. From 2004 to 2017, many studies showed that *L. chuanxiong* is seriously threatened by Cd pollution, and approximately 87.99% of *L. chuanxiong* experienced Cd pollution incidents (Han et al., 2008; Zhang et al., 2019; Zhou et al., 2021; Yao et al., 2022). As a Cd enrichment plant, the enrichment capacity of Cd and Pb in the underground rhizomes of *L. chuanxiong* was 1351 times that of Cr (chromium), 320 times that of lead, 417 times that of As (arsenic), and 18.5 times that of Hg (mercury) (Yi and Peng, 2007). Additionally, the serious threat posed to *L. chuanxiong* by Cd pollution has also caused economic difficulties for local farmers, which has, in turn, introduced great obstacles to the development of the rural economy (Zhang et al., 2019). Therefore, to establish a comprehensive evaluation and planning system for important cash crops, we chose *L. chuanxiong* as an illustrative case and determined its priority planting areas based on current and future conditions.

Here, we develop current and future climatic niche models of *L. chuanxiong* and a spatiotemporal scenario prediction model for soil Cd pollution to identify suitable cultivation regions and areas protected from soil pollution threats. The objectives of this study include the following: (1) establishing a comprehensive evaluation framework for soil pollution by heavy metals and cash crops; (2) planning the priority planting area for *L. chuanxiong* based on current conditions*;* and (3) simulating the spatial dynamics of the planned regions and determining future priority planting areas for *L. chuanxiong*.

## 2 Materials and methods

### 2.1 Data source

#### 2.1.1 Collection of spatial data

**Figure 1:**
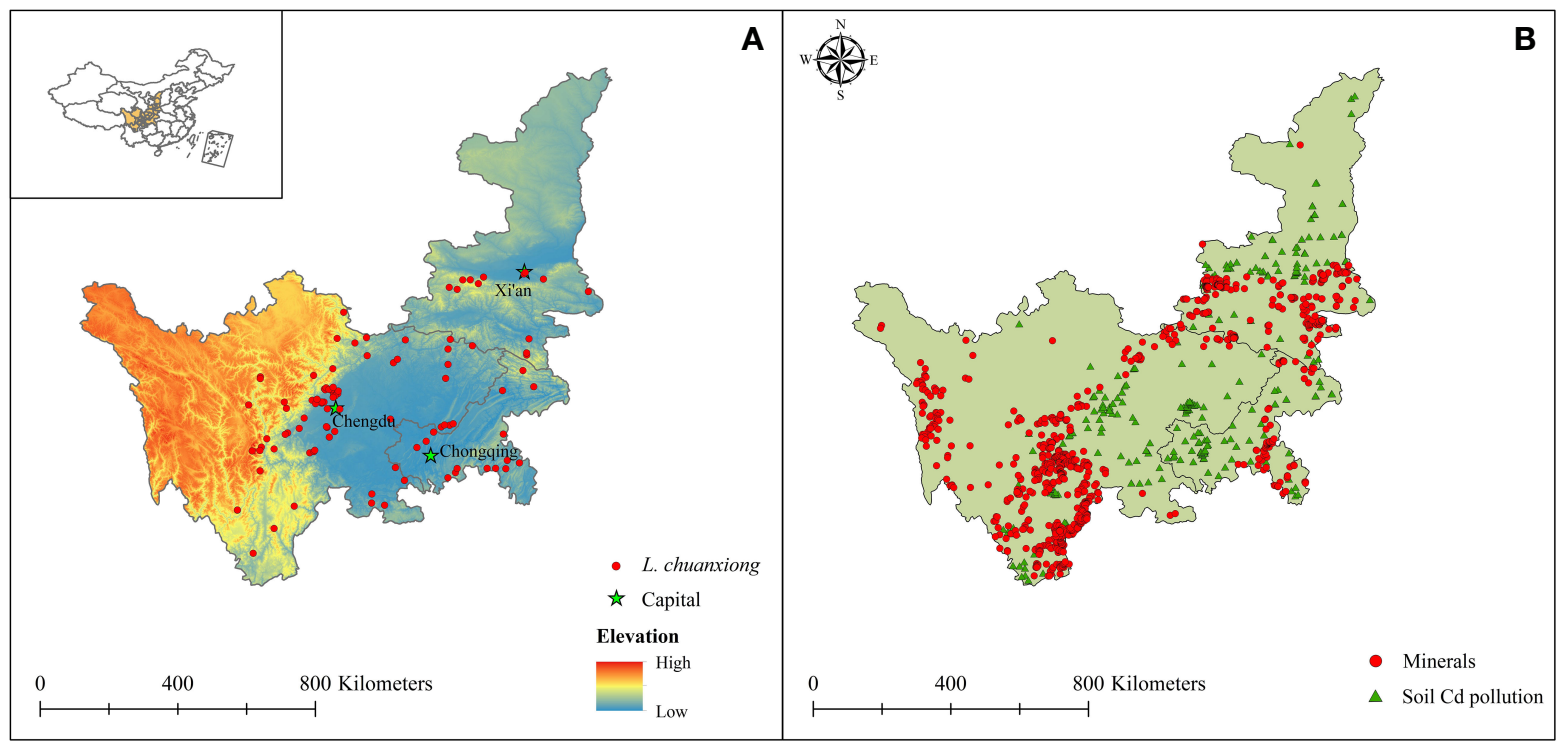
Distribution records of *L. chuanxiong*, minerals, and soil Cd pollution data in the study area. **(A)** The occurrence of *L. chuanxiong* and elevation in the study area; **(B)** The minerals are the latitude and longitude distribution points of the mining area, including Cd, Pb and Zn mines; the soil Cd pollution represents the sampling point of the soil Cd content collected from the literature.

*L. chuanxiong*, a significant economic plant and medicinal crop in Chinese, medicine is mainly distributed in western and southwestern China and is especially concentrated in Sichuan Province and Chongqing city (Fang et al., 2020; Ni et al., 2021). After comprehensive consideration, three provinces (Sichuan, Shaanxi, and Chongqing) were selected to constitute the study area; we conducted an extensive literature search and accessed a large number of online databases, such as the Plant Photo Bank of China (http://ppbc.iplant.cn/), the Global Biodiversity Information Facility (https://www.gbif.org/), the Specimen Resources Sharing Platform for Education (http://mnh.scu.edu.cn/), and the Chinese Virtual Herbarium database (http://www.cvh.ac.cn/), to obtain more accurate distribution data for *L. chuanxiong*. We collected 30 years (1990-2020) of spatial distribution data for *L. chuanxiong*. For this part, spatial occurrence data without accurate location information were removed, and if the exact geocoordinates of the occurrence records were not available, Google Earth (http://ditu.google.cn/) was used based on geographical location descriptions to identify latitude and longitude (Wei et al., 2018). In this study, due to the similar ecological characteristics in areas of 1 km^2^, ArcGIS 10.3 was used to filter duplicate occurrence data in each grid cell (1 km × 1 km), and only one point was retained (Khanum et al., 2013). Finally, a total of 103 spatial distribution data points were ultimately obtained (**Figure 1A**) and saved in “CSV” format in accordance with the requirements of the MaxEnt model, which is the most widely used niche model. This model has abundant advantages, such as a short run time, operational ease, good performance, and high precision (Elith et al., 2011; Radosavljevic & Anderson, 2014).

### 2.1.2 Environmental parameters

To accurately construct a climate niche model and predict the current spatial geographical distribution of *L. chuanxiong*, the habitat characteristics of the species were comprehensively considered. The final environmental parameters were from four categories (bioclimate, soil, topography, and others), and a total of 46 variables affecting the growth and distribution of *L. chuanxiong* were considered (**Table 1**). According to the source of the data, 19 bioclimatic variables, 3 terrain variables, 1 vapor pressure variable, 1 wind variable, and 1 solar radiation variable were obtained from the World Climate Database (www.worldclim.org), and 2 terrain variables were extracted from elevation data, including slope and aspect. Furthermore, 19 soil variables were obtained from the Harmonized World Soil Database (https://webarchive.iiasa.ac.at/Research/LUC/External-World-soil-database/), including 14 surface soil parameters and 5 soil quality factors. Land cover and population density data were collected from the Socioeconomic Data and Applications Center (https://sedac.ciesin.columbia.edu/).

**Table 1:** Basic information for the current environmental variables.

Category	Variable	Description	Unit	Resolution
Bioclimate	Bio1	Annual Mean Temperature	°C×10	1 km
Bioclimate	Bio2	Mean Diurnal Range	°C×10	1 km
(http://www.worldclim.org/)	Bio4	Temperature Seasonality	/	1 km
	Bio7	Temperature Annual Range	/	1 km
	Bio12	Annual Precipitation	mm	1 km
	Bio13	Precipitation of Wettest Month	mm	1 km
	Bio14	Precipitation of Driest Month	mm	1 km
	Bio15	Precipitation Seasonality	/	1 km
Soil	Soil1	Topsoil Base Saturation	%	1 km
Harmonized World Soil Database	Soil2	Topsoil Calcium Carbonate	%weight	1 km
(https://webarchive.iiasa.ac.at/Research/LUC/External-World-soildatabase/)	Soil3	Topsoil Gypsum	%weight	1 km
	Soil4	Topsoil CEC-clay	cmol/kg	1 km
	Soil5	Topsoil CEC-soil	cmol/kg	1 km
	Soil6	Topsoil Clay Fraction	%wt	1 km
	Soil7	Topsoil Salinity-Elco	dS/m	1 km
	Soil8	Topsoil Sodicity-ESP	%	1 km
	Soi19	Topsoil Gravel Content	%vol	1 km
	Soil10	Topsoil Organic Carbon	%weight	1 km
	Soil11	Topsoil pH-H2O	-log(H^+^)	1 km
	Soil12	Topsoil Sand Fraction	%wt	1 km
	Soil13	Topsoil Silt Fraction	%wt	1 km
	Soil14	Topsoil Teb	cmol/kg	1 km
	Soil15	Nutrient Availability	/	1 km
	Soil16	Nutrient Retention Capacity	/	1 km
	Soil17	Rooting Conditions	/	1 km
	Soil18	Oxygen Availability to Roots	/	1 km
	Soil19	Excess Salts	/	1 km
Topography	Altitude	/	m	1 km
WorldClim database	Aspect	/	/	1 km
(http://www.worldclim.org/)	Slope	/	°	1 km
Other	Wind	/	m/s	1 km
WorldClim database	Den	Population Density	/	1 km
(http://www.worldclim.org/)	Ter	Land Cover	/	1 km
Socioeconomic Data and Applications Center	Srad	Solar Radiation	kJ/m^2^/day^1^	1 km
(https://sedac.ciesin.columbia.edu/)	Vapr	Vapor Pressure	kPa	1 km

Since the resolution, projection, and range of all environmental variables were mostly consistent, the resolution of all environmental factors was adjusted to 1 km (approximately 30 s), the projection was GCS_WGS_1984, and the raster layer range of the environmental factors was clipped to the size of the study area (Phillips and Dudík, 2008). To avoid overfitting due to strong correlations between environmental variables in the final modeling results, the 19 bioclimate factors were filtered using Pearson’s correlations in SPSS; in each step, a factor that was highly significantly related to the distribution of *L. chuanxiong* was retained and a factor with a correlation coefficient ≥ 0.8 was eliminated (Yang et al., 2013; Cao et al., 2016). Ultimately, 35 environmental variables that influence the distribution of *L. chuanxiong*, including 8 bioclimate variables and 19 soil factors (**Table 1**), were derived to perform spatial niche modeling in combination with occurrence point data. The details of the categories, names, and units of all the environmental parameter data are presented in **Table 1**.

For the future climate scenarios, all environmental parameters used to construct the niche model were consistent with the current values except for the bioclimate factors, which reflected four representative concentration pathways (RCPs) in the Coupled Model Intercomparison Project Phase 6 (CMIP6) (Liu et al., 2019; Anibaba et al., 2022). These four RCPs (RCP2.6, RCP4.5, RCP6, and RCP8.5) represent different climate change states (from the lowest emission scenario to the highest emission scenario) and can be applied to research on sustainable development and biodiversity changes (Meinshausen et al., 2011; Chong-Hai and Ying, 2012; Legg, 2021). Specifically, two time periods (2041-2060 and 2081-2100) were considered in our study, and four RCPs indicated total radioactive forcing values of +2.6, +4.5, +6.0 and +8.5 W/m^2^ in 2100 (IPCC, 2014; Zhang Y. et al., 2016). We collected bioclimatic prediction data from the World Climate Database (www.worldclim.org) under 8 climate change scenarios (2050-RCP2.6, 2050-RCP4.5, 2050-RCP6.0, 2050-RCP8.5, 2070-RCP2.6, 2070-RCP4.5, 2070-RCP6.0, and 2070-RCP8.5). Then, we acquired 8 bioclimatic parameters for each climate scenario (Bio1, Bio2, Bio4, Bio7, Bio12, Bio13, Bio14, and Bio15) using ArcGIS 10.3, and the remaining 27 variables (soil, topography, etc.) were unchanged. In short, we obtained 8 climate change datasets through elaborate processing for future climate niche predictions combined with occurrence point data. The detailed information regarding future climate scenario factors was consistent with current information.

### 2.1.3 Soil Cd pollution and mineral distribution data

Planning the priority planting area is conducive not only to expanding the distribution of *L. chuanxiong* but also to avoiding the threat of soil Cd pollution. By consulting many documents, 432 soil Cd pollution data points in the study area were collected (**Figure 1B**), including accurate spatial geographic coordinates and soil Cd content values. The spatial interpolation method was used to calculate the soil Cd pollution status based on the interpolation of Cd content values at known points in space to evaluate the spatial characteristics of soil Cd pollution in the study zones (Ma et al., 2018; Sheng et al., 2021). Studies found that because the associated minerals of Pb and Zn ore contain Cd ore and a large amount of Cd pollution occurs in the soil surface of regions with Pb and Zn ore, Pb and Zn ore are also considered when selecting mineral data (Xiao et al., 2017; Tepanosyan et al., 2018; Qiao et al., 2019). The mineral data were obtained from the National Geological Archives (http://www.ngac.org.cn/), including 997 longitude and latitude points for Cd, Pb and Zn mines (**Figure 1B**). In this study, the impact of mining areas on the surrounding soil environment was assessed by using nuclear density analysis (Xiao and Fu, 2020; Hou and Zheng, 2021; Zou et al., 2022).

### 2.2 Methods

#### 2.2.1 Methodology overview

**Figure 2:**
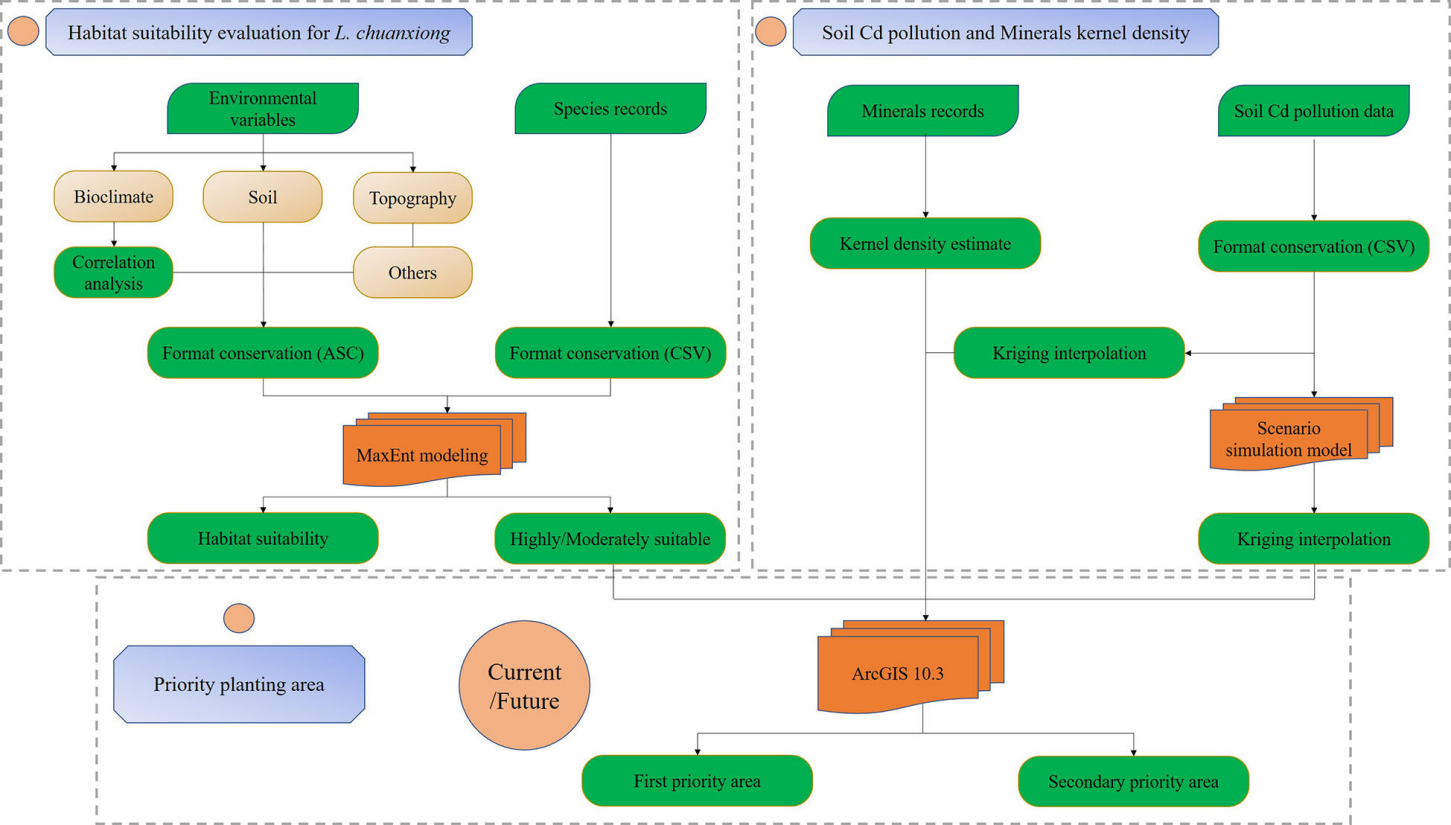
Research framework.

In this study, the MaxEnt model was used to fit the spatial distribution of *L. chuanxiong* using environmental parameters and geographical occurrence data. Then, we used geographic information technology, including the spatial interpolation method and nuclear density analysis, to perform spatial interpolation and mine impact analysis (Zhao et al., 2016; Sun et al., 2023). By coupling the results of the niche model, soil Cd pollution, and mine impact analysis, the priority areas for *L. chuanxiong* were identified to infer the best areas for *L. chuanxiong* under current climate conditions. To further explore the spatial dynamic changes in priority zones for *L. chuanxiong* caused by a series of processes, we developed a climate niche model and a spatiotemporal scenario prediction model for soil heavy metals to simulate this change (Wu, 2008; He et al., 2020; Tang et al., 2021; Hou et al., 2022). The specific methods included the following steps: (1) performing MaxEnt modeling to evaluate habitat suitability for *L. chuanxiong*; (2) planning priority areas of *L. chuanxiong* on the basis of the MaxEnt model, soil Cd pollution, and mine impact analysis; and (3) analyzing the changes in planned areas using climate niche and spatiotemporal scenario prediction models (**Figure 2**)

#### 2.2.2 Habitat suitability evaluation

Human activities and other factors, such as logging, livestock grazing, natural disasters, and interspecific interactions, were assumed to have no influence. Incorporating these assumptions, MaxEnt 3.4.0 software (Phillips, 2005) was used to project the spatial geographic distribution of *L. chuanxiong* using the species occurrence data and environmental factors. We hypothesized that the bioclimatic variables would have excellent performance due to their indispensable roles in the metabolic processes and distributions of vegetation (Gao et al., 2018). We compared various modeling results by adjusting the parameters of the model to enhance model robustness, including the maximum number of iterations, random test ratios, maximum number of background points, and convergence threshold. Finally, the parameter settings of the MaxEnt program executed in this study were as follows: the maximum number of iterations was set to 1000, 30% of the location point data of *L. chuanxiong* were randomly selected as the test set, the remaining data comprised the training set, the convergence threshold was set to 10^–5^, the maximum number of background points was equal to 10,000, and the average results of 10 replicates were considered the final outcome. The output format was set as “ASC”, a logistic output format, and the other formats were set to the software defaults (Remya et al., 2015). The jackknife method was utilized in our study to determine the significance of different variables, and corresponding response curves were created to obtain the suitable distribution range for *L. chuanxiong* (He et al., 2021).

The accuracy of MaxEnt modeling was evaluated using the area under the receiver operating characteristic (ROC) curve (AUC) (Phillips and Dudík, 2008; Venne and Currie, 2021). In general, an AUC value > 0.9 indicates excellent performance, values between 0.8 and 0.9 indicate good model performance, values between 0.7 and 0.8 indicate average performance, and values below 0.7 indicate poor performance (Du, 2018). The output results were visualized, and habitats suitable for *L. chuanxiong* were mapped using the reclassify tool in ArcGIS 10.3. The potential suitable habitats of *L. chuanxiong* were divided into four categories: highly suitable (0.6–1), moderately suitable (0.4–0.6), minimally suitable (0.2–0.4), and not suitable (0–0.2) (Moreno et al., 2011; Ma and Sun, 2018).

#### 2.2.3 Planning the priority planting areas for *L. chuanxiong*

To further identify ecological cultivation areas of *L. chuanxiong* without the influence of soil Cd pollution, we fully considered the suitable niche of the species and coupled it with the background values of soil Cd and the impact of mining areas to determine high-quality planting regions. Generally, kriging interpolation is a spatial method for unbiased optimal estimation of regional variables based on variogram theory and structural analysis, including simple kriging (SK), universal kriging (UK), synergistic kriging (CK), and ordinary kriging (OK) (Oliver and Webster, 1990; Van Beers and Kleijnen, 2004). In this study, OK was used to spatially interpolate soil Cd content values, which is conducive to simulating the spatial and geographic variation trends of Cd pollution (Gia Pham et al., 2019; Munyati and Sinthumule, 2021). The specific formula is as follows:


(1) $$Z^{*}(X_{0})=\sum {\;}_{i=1}^{n}\lambda_{i}Z(x_{i})$$

Where *Z* ^*^(*X*_0_) represents the estimated value of the soil Cd content at a certain point, *λ_i_* is the weighting coefficient, which represents the weight of sites included in the interpolation on the estimated soil Cd content, and *x_i_ Z*(*x_i_*) represent the location of the measured site and the value of the soil Cd content at the site, respectively. Incorporating the results of soil Cd interpolation, the Reclassify tool of ArcGIS 10.3 was used for processing, and the corresponding raster layer was extracted in accordance with the first level of national standards (0<Cd ≤ 0.2 mg·kg^-1^) and second level of national standards (0.2 mg·kg^-1^<Cd ≤ 0.3 mg·kg^-1^) (Zheng, 2003; Ministry of Environmental Protection and Ministry of Land and Resources of China, 2014).

Kernel density estimation, a nonparametric spatial analysis method for calculating the density of spatial features in their surrounding neighborhoods, is widely used to simulate the kernel density distribution in space (Silverman, 1981; Zambom and Ronaldo, 2013). This method can accurately reflect the spatial aggregation degree and changing trend of research elements; the closer to the center these factors are, the greater the impact will be, and vice versa (Okabe et al., 2009). The specific formula is as follows:


(2) $$f(x)=\frac{1}{nh}\sum {\;}_{i=1}^{n}k(\frac{x-x_{i}}{h})$$

where *n* is the number of sample points, *k* is the weight kernel function, *h* represents the threshold of the range, and *x*–*x_i_* represents the distance from the preestimated point of *x* to the sample point of *x_i_*.

Accordingly, highly and moderately suitable areas were extracted on the basis of the results of niche modeling and overlaid with the first and second levels of national standards for soil Cd pollution, respectively. The final priority planting areas were identified by excluding areas with high mining influence from the resulting region.

#### 2.2.4 Spatial dynamic changes in priority planting areas of *L. chuanxiong*

Identifying the future priority planting zones for *L. chuanxiong* under climate change is a prerequisite for simulating the dynamic spatial changes in priority planting areas of this species. First, MaxEnt modeling projections were obtained for eight future climate change scenarios to predict the potential habitat distribution of *L. chuanxiong* in the future (Kumar, 2012). Highly and moderately suitable areas were selected using the same classification method as the current conditions to classify suitability levels.

Then, we utilized a scenario simulation model of soil heavy metals to simulate the prediction value in the study region (Zhang J. et al., 2016). From the perspective of dynamic equilibrium, the temporal prediction of soil pollution by heavy metals must be based on their dynamic changes at known temporal scales, and the input and output of heavy metals are two key parameters in the model (Wu, 2008). Therefore, the scenario simulation model used in this study is very suitable for predicting changes in soil Cd pollution in 2050 and 2070 based on the current data. In this case, the future concentration of soil Cd is:


(3) $$Q_{t}=Q_{0}K^{t}+QK(1-K^{t})/(1-K)$$

where *Q_t_* is the content of Cd in the soil after *t* years (mg/kg), *Q*_0_ is the initial content of Cd in the soil (mg/kg), *Q* is the annual content of Cd in the soil (mg/kg), *K* is the annual residual rate of soil Cd (0.96) (Lin, 2009), and *t* represents the years of prediction (year).

To quantify the change dynamics of soil Cd pollution, we utilized three scenario prediction models to calculate the content of soil Cd at some time in the future, including the optimistic scenario, default scenario, and pessimistic scenario (Wu, 2008; He et al., 2020). In the optimistic scenario, for an extended period, no heavy metal ions will enter the pedosphere after the discharge of heavy metal emissions from factories, businesses, transportation, and residential sewage has been processed. In the default scenario, factories and businesses responsible for heavy metal emissions follow usual production, and discharges such as those from transportation and residential sewage remain unchanged according to the current situation. In the pessimistic scenario, the population grows, urbanization accelerates, and the number of businesses and factories responsible for heavy metal emissions increases, ultimately leading to a continuation of heavy metal ions entering the soil. The specific formulas are as follows.

Optimistic scenario:


(4) $$Q_{t}=\{\begin{array}{cc} C_{0}K^{t} & C_{0}>C_{b}\\ C_{0} & C_{0}\leq C_{b} \end{array}$$

Default scenario:


(5) $$Q_{t}=\{\begin{array}{cc} Q_{0}K^{t}+VK\frac{1-K^{t}}{1-K} & Q_{0}>Q_{b}\\ Q_{0} & Q_{0}\leq Q_{b} \end{array}$$

Pessimistic scenario:


(6) $$Q_{t}=\{\begin{array}{cc} Q_{0}K^{t}+VK\frac{1-K^{t}}{1-K}+aK\sum {\;}_{m=1}^{n}K^{n-m} & Q_{0}>Q_{b}\\ Q_{0} & Q_{0}\leq Q_{b} \end{array}$$

where *Q_b_* is the background value of soil Cd in the study zone (0.85 mg/kg). Since the soil Cd background values of the three provinces are different, we obtained the background value Cd content by calculating the mean and retained two decimal places.

In addition, we referred to the average publication years of the papers presenting the data on soil Cd pollution that were collected in this study based on a literature review, regarding soil Cd content in 2018 as the initial value. For *Q*, the annual content of Cd in the soil was assigned according to the first year of “zero pollution”, and the first year was selected in accordance with the economic development of the study area (Wu, 2008). Since China underwent reform and opened up, the southwest region has experienced vigorous industrial and agricultural development, resulting in increasingly serious environmental problems (Yan, 2016). Before 1978, there were no significant reports of soil contamination incidents in the study zone (Yan, 2016). Therefore, 1978 was set as the first year of soil Cd pollution to calculate the annual content of Cd in the soil over a period spanning 40 years. After obtaining the value of the soil Cd content for every geographical coordinate in the study area in 2050 and 2070, OK interpolation was performed to simulate the spatial and geographic variation trends of Cd pollution. Finally, we used a similar method to determine the regions meeting the first level and the second level of national standards. In this study, the influence of the kernel density estimate for minerals was assumed to be unchanged. The future priority planting areas for *L. chuanxiong* were determined in a similar way.

## 3 Results

### 3.1 Spatial niche model

#### 3.1.1 Contribution of environmental variables

In this study, a spatial niche model for *L. chuanxiong* based on species distribution data and environmental parameters was developed, and habitat suitability maps were obtained by using ArcGIS 10.3. Overall, the AUC training values for *L. chuanxiong* indicate that the model performed quite well when current and future niche modeling was conducted (**Figures S1-S3**).

**Figure 3:**
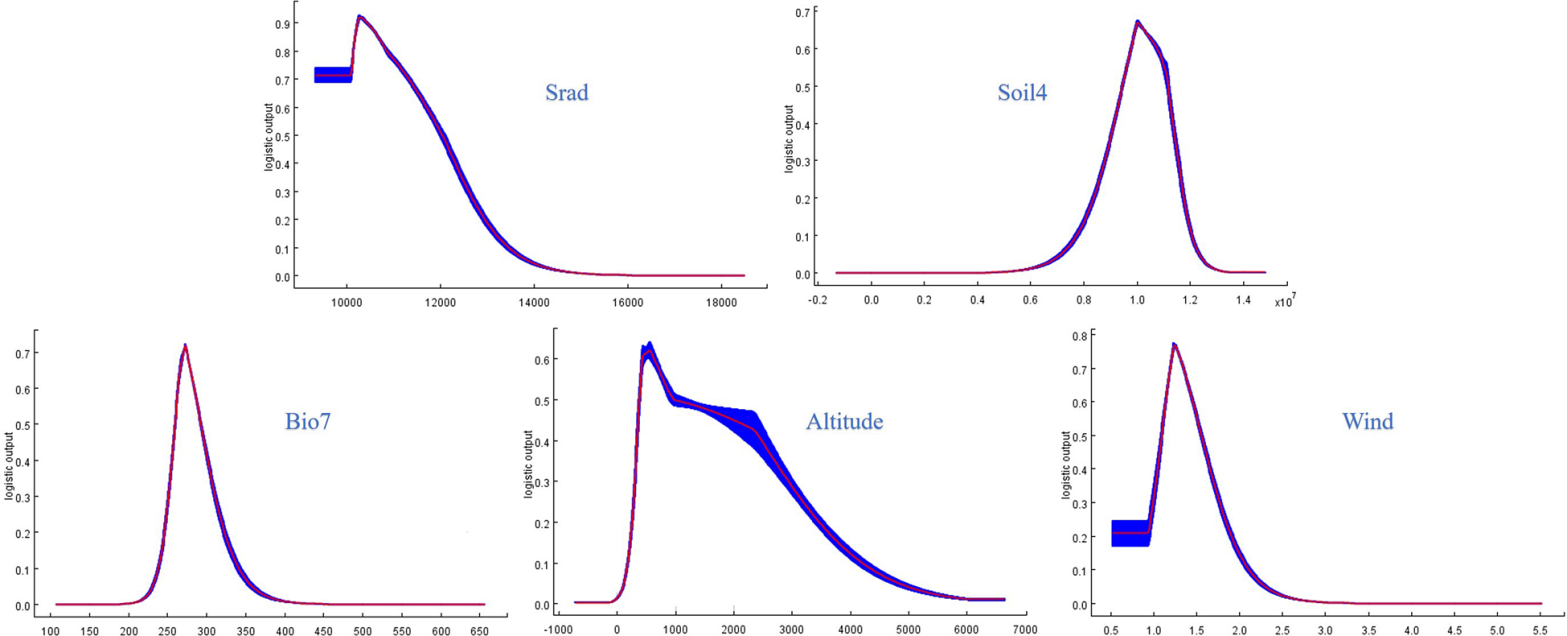
Critical environmental parameters included in the current MaxEnt model. Srad, Soil4, and Bio7 represent the solar radiation, topsoil cec clay, and annual temperature range, respectively.

Key environmental parameters, an important part of niche modeling, are drivers of the current distribution pattern of *L. chuanxiong* and are fundamental for predicting the changes in future distribution patterns. The average contribution rates of every environmental factor were determined by the current spatial niche model to obtain the critical environmental parameters. Overall, the results revealed that the dominant factors for *L. chuanxiong* were solar radiation (Srad, 55.4%), topsoil cec-clay (Soil4, 19.9%), temperature annual range (Bio7, 4.8%), altitude (4%), and wind (3.8%), together explaining 87.9% of the variation. These different environmental parameters belong to various categories and indicate the comprehensive influence of variables on *L. chuanxiong*, including its spatial geographical distribution and growth. The optimal ranges for *L. chuanxiong* were determined by using the response curves of important environmental variables. According to the corresponding response curves, the average daily solar radiation was not higher than 12028.92 kJ·m^-2^, topsoil cec-clay ranged from 0.95 cmol/kg to 1.12 cmol/kg, the annual temperature range was between 25.98 and 29.23°C, the altitude ranged from 930.31 to 974.54 m, and the most suitable range for wind was 1.09~1.55 m/s (**Figure 3**).

#### 3.1.2 Spatial distribution of *L. chuanxiong*

**Figure 4:**
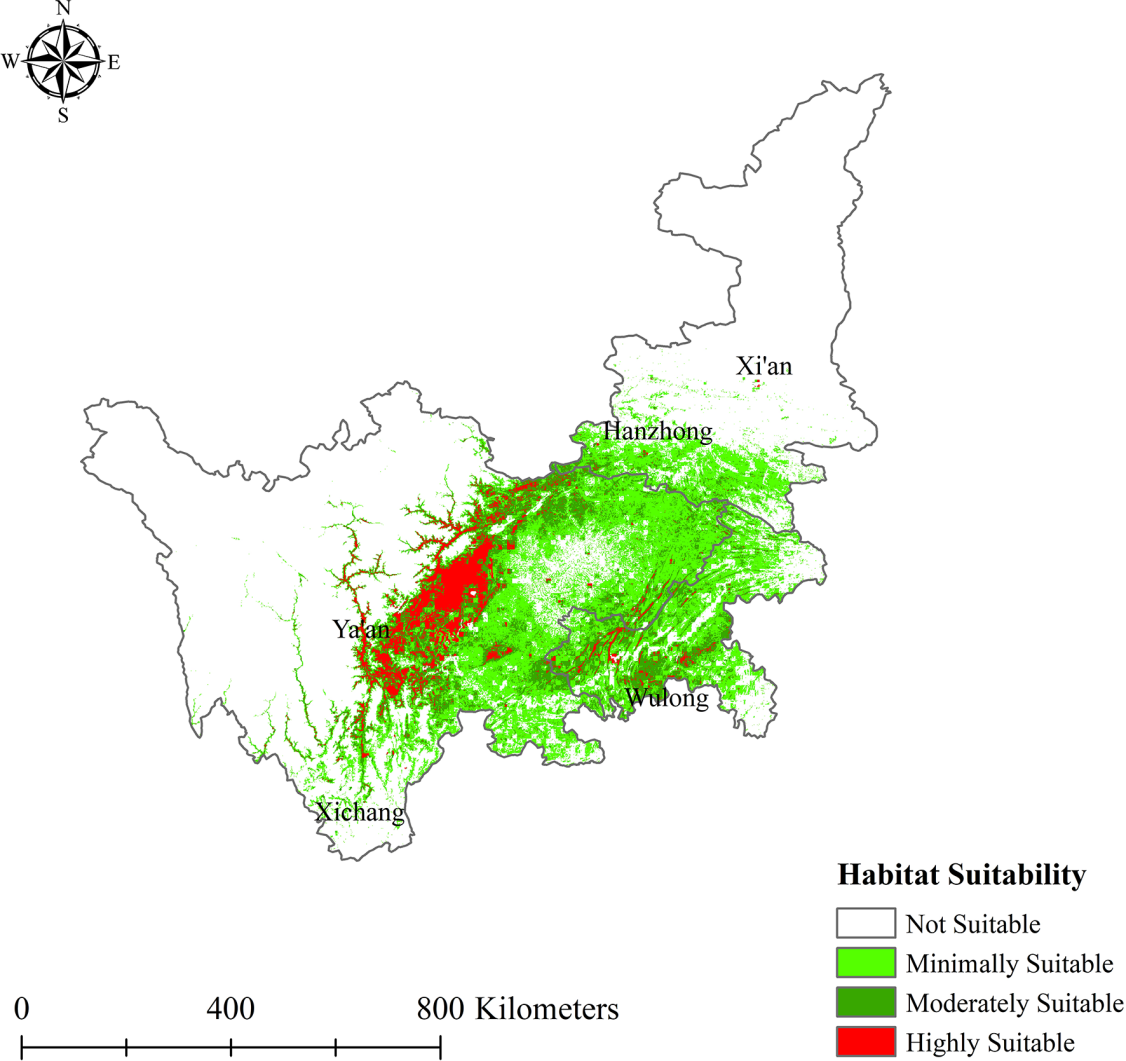
Current potential distribution of *L. chuanxiong*.

In general, the current suitable habitat areas for *L. chuanxiong* were mainly distributed in central and eastern Sichuan, southern Shaanxi, and parts of Chongqing, with an area of approximately 3.28×10^5^ km^2^. The results of MaxEnt modeling indicated that habitat suitability was too broad, the coverage area was very large, and the areas of high and moderate suitability were mainly concentrated in Chengdu, gradually decreasing from north to south (**Figure 4**). The highly suitable areas were concentrated on the Chengdu Plain, extending to the junction of Ya’an and Xichang in the south and Guangyuan in the north and covering Chengdu, Meishan, Deyang, and Ya’an. Additionally, there was a sporadic distribution in Leshan, Mianyang, Xichang, Guangyuan, Hechuan, Wulong, and other regions, with an area of 0.37×10^5^ km^2^, corresponding to 4.72% of the study area. The moderately suitable areas accounted for 11.28% of these habitats and are approximately 0.87×10^5^ km^2^ in total area, including portions of Guanyuan, Hechuan, Wulong, and Leshan.

**Figure 5:**
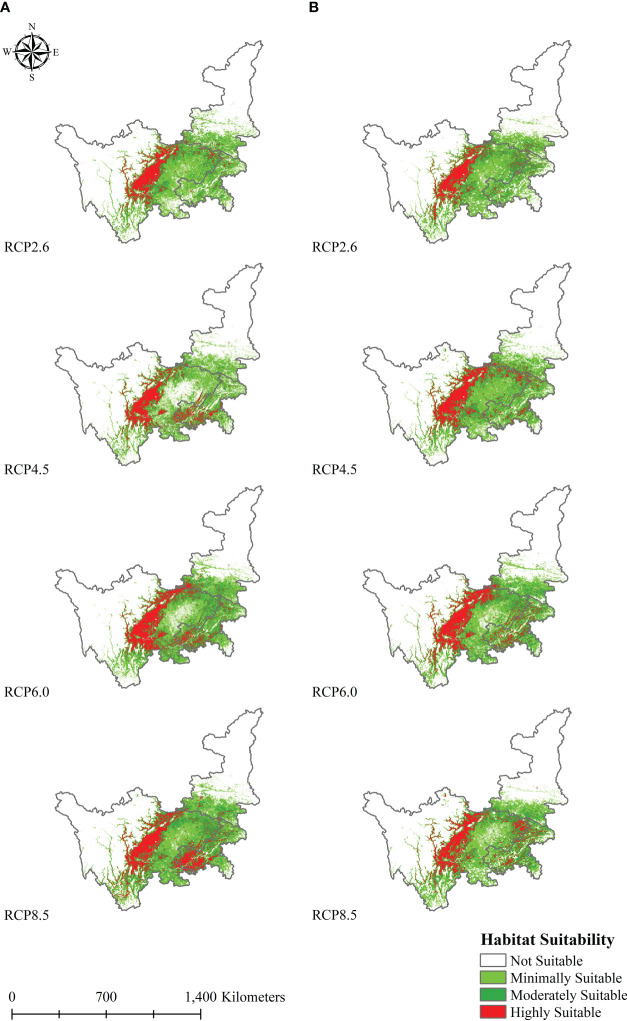
Future potential distribution of *L. chuanxiong*; **(A, B)** represent 2050 and 2070, respectively.

For different climate scenarios, in this study, the Reclassify tool was used to process the output results of the climate niche model with the same division criteria. **Figure 5** and **Table 2** show the spatial distribution variation trends and specific values for suitable regions. According to future predictions, the suitable area for *L. chuanxiong* was mainly distributed in central and eastern Sichuan, southern Shaanxi, and Chongqing; some areas, such as Suining and Nanchong, fluctuate greatly. The model results suggest that highly and moderately suitable habitat regions have similar distributions, and the change trend is the same as that under current climate conditions. In 2050, the largest and smallest distribution areas of *L. chuanxiong* were observed for the RCP8.5 climate scenario (4.35×10^5^ km^2^) and RCP8.5 climate scenario (3.34×10^5^ km^2^), respectively. For highly and moderately suitable areas, the largest distribution occurred assuming the RCP8.5 climate scenario, followed by the RCP6.0, RCP4.5, and RCP2.6 climate scenarios. In 2070, the largest distribution areas were observed for high and moderate suitability under the RCP 6.0 climate change scenario, followed by the RCP 8.5 climate scenario.

**Table 2:** Distribution of *L. chuanxiong* suitable areas 486 under current and future conditions.

Climate change	Suitability level					
	High (×10^5^ km^2^)	Proportion (%)	Moderate (×10^5^ km^2^)	Proportion (%)	Minimal (×10^5^ km^2^)	Proportion (%)
Current	0.37	4.7	0.87	11.3	2.05	26.4
2050-RCP2.6	0.64	8.3	1.04	13.5	2.05	26.5
2050-RCP4.5	0.73	9.4	0.92	11.9	1.69	21.8
2050-RCP6.0	0.93	12.1	1.39	18.0	1.63	21.0
2050-RCP8.5	1.01	13.0	1.54	19.9	1.8	23.3
2070-RCP2.6	0.64	8.3	1.12	14.5	1.86	24.0
2070-RCP4.5	0.85	10.9	1.25	16.1	1.87	24.1
2070-RCP6.0	0.94	12.3	1.30	16.8	1.72	22.2
2070-RCP8.5	0.8	10.3	1.29	16.6	1.82	23.5

### 3.2 Current priority planting sites for *L. chuanxiong*

**Figure 6:**
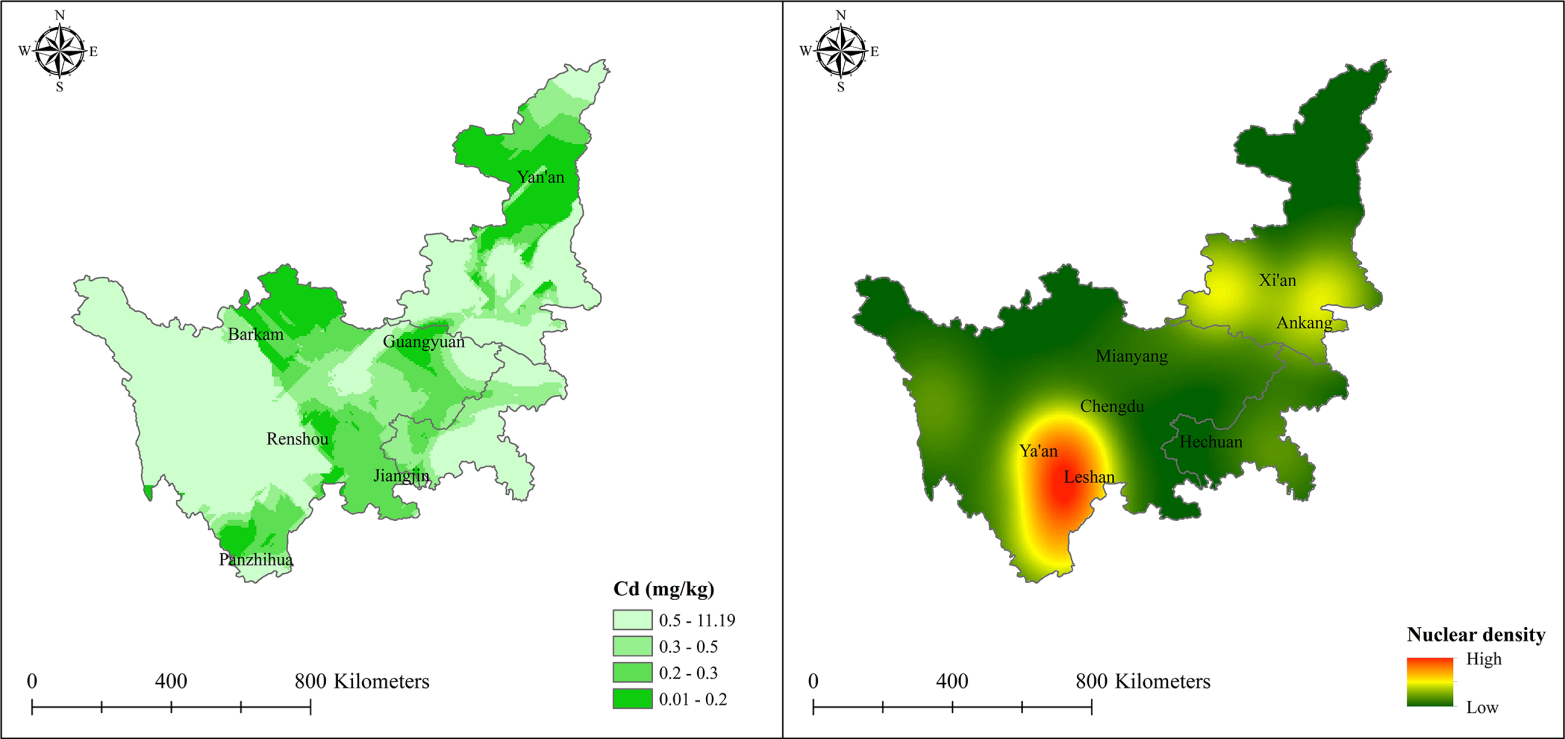
Current soil Cd pollution and mineral nuclear density influence.

The results of OK interpolation of the soil Cd content showed that the areas including Meishan, Guangyuan, southern Chengdu, western Yibin, and northern Leshan all met the national first-level standard for soil Cd pollution (**Figure 6**). Simultaneously, some areas, such as Hanzhong, Guangyuan, and Xichang, also have extremely low soil Cd pollution. The areas that meet the national secondary standard for soil Cd pollution were mainly concentrated in Ziyang, Neijiang, Zigong, Yibin, Luzhou, and other places. Additionally, the soil Cd pollution in Dujiangyan, Chongzhou, Pixian, Wenjiang, Pingwu, Jiangyou, and other regions was extremely insignificant. Panzhihua city, Xichang city, and other regions examined in our study in southern Sichuan were less affected by soil Cd pollution and met the national standards, which is beneficial for the cultivation of *L. chuanxiong*.

To further evaluate the influence of minerals in the study zone, Cd, Pb and Zn mines were included in the kernel density estimation; a darker color indicates a stronger degree of influence for the mineral (**Figure 6**). The distribution of minerals in Xichang, Ya’an, and Leshan was relatively dense, and more planning or conservation measures should be taken when planting *L. chuanxiong* in these regions because of the greater impact of minerals. Sichuan, Tianquan, Hanyuan, Hongya, Danling, and other counties were within the zone of relatively high mineral influence, while Pengshan and Renshou Counties were less affected. In Shaanxi, most regions were safe, except for a slight influence of soil Cd pollution in Hanzhong, Shangzhou, Ankang, Xi’an, and Shiyan.

**Figure 7:**
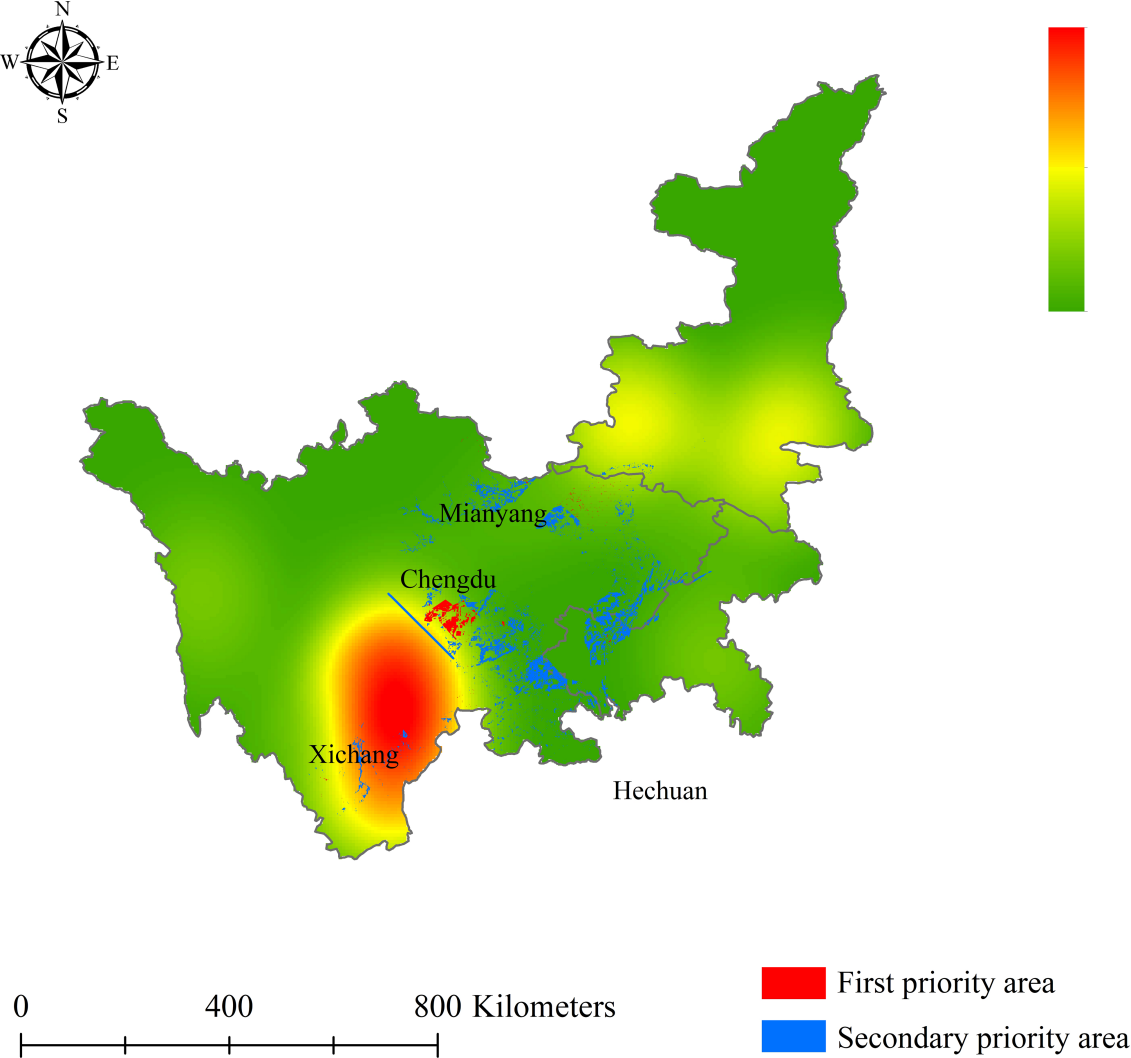
Current priority planting area for *L. chuanxiong*.

Ultimately, the priority planting zones for *L. chuanxiong* were obtained by superimposing the highly and moderately suitable areas with the first and secondary soil Cd pollution levels, respectively, under the context of mineral influence (**Figure 7**). More specifically, the first-priority planting area for *L. chuanxiong* originated from a combination of highly suitable zones determined by the MaxEnt model and the first standard soil Cd pollution areas; secondary-priority planting areas were also identified by these methods. As such, the first-priority planting zones are the most suitable areas for the distribution and growth of *L. chuanxiong*, while the division of secondary-priority planting areas ensures the yield of cash crops, thereby contributing to artificial cultivation planning and management for relevant leaders. Based on the current climate scenario, the first-priority areas are mainly distributed in southern Chengdu and northern Meishan, including Meishan, Pujiang, Qionglai, Pengshan, Xinjin, Renshou, and other counties. In total, the spatial distribution areas for first and secondary planting are 2.06 ×10^3^ km^2^ and 1.64 ×10^4^ km^2^, respectively. According to the results of the distribution areas for priority cultivation zones, the areas of Meishan County (0.58 ×10^3^ km^2^), Qionglai County (0.33 ×10^3^ km^2^), Pujiang County (0.25 ×10^3^ km^2^), Pengshan County (0.18 ×10^3^ km^2^), Xinjin County (0.15 ×10^3^ km^2^), Renshou County (0.14 ×10^3^ km^2^), and Dayi County (0.11 ×10^3^ km^2^) exceeded 100 km^2^ (**Table 3**). In the secondary-priority planting zone, a total of twelve counties were planned, with areas exceeding 400 km^2^. Among them, Pingwu County was found to be the best region in the secondary-priority planting zone, accounting for 6.84% of the total area, followed by Lu County (0.98 ×10^3^ km^2^, 5.71%) and Hechuan County (0.88 ×10^3^ km^2^, 5.14%). Other counties, such as Jiange, Longchang, Zizhong, and Fushun, are now also recognized as excellent cultivation regions, which is conducive to increasing the production of *L. chuanxiong* (**Table 3**). The complete priority planting area can be viewed in **Tables S2** and **S3**.

**Table 3:** Current priority planting area for *L. chuanxiong*, including the seven first-priority counties and twelve secondary-priority counties.

Priority area	County	Area (×10^3^ km^2^)	Proportion (%)
First	Meishan	0.58	28.0
	Qionglai	0.33	16.0
	Pujiang	0.25	12.4
	Pengshan	0.18	8.8
	Xinjin	0.15	7.5
	Renshou	0.14	6.5
	Dayi	0.11	5.3
Secondary	Pingwu	1.11	6,5
	Lu	0.98	5.7
	Hechuan	0.88	5.1
	Jiange	0.86	5.0
	Longchang	0.69	4.0
	Fushun	0.53	3.1
	Weiyuan	0.49	2.8
	Jiangbei	0.48	2.8
	Zizhong	0.46	2.7
	Ziyang	0.45	2.6
	Renshou	0.44	2.6
	Yuechi	0.4	2.4

### 3.3 Future priority planting sites for *L. chuanxiong*

**Figure 8:**
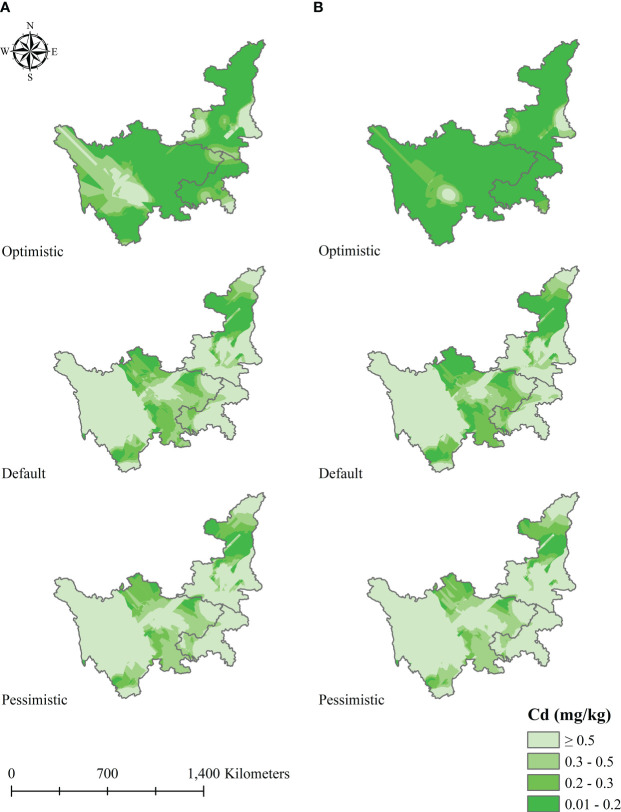
Future soil pollution in the study area obtained by the scenario simulation model; **(A, B)** represent 2050 and 2070, respectively.

To obtain the first- and secondary-priority planting areas, we utilized three scenario prediction models and OK interpolation to simulate the spatial dynamics of soil Cd pollution in 2050 and 2070. Overall, a background map of soil Cd pollution in the study area was generated using the analysis and mapping tool of the geographic information system in accordance with the result of OK interpolation (**Figure 8**). The soil Cd pollution areas with the greatest changes were mainly concentrated in central and northeastern Sichuan, including Chengdu, Deyang, Meishan, Leshan, Guangyuan, and Nanchong. Based on three soil Cd pollution scenarios, the soil Cd pollution within the study area in 2050 and 2070 varied widely, which explains the difference in economic development status in the future. Soil Cd pollution continually improved under the optimistic scenario, but the changes were not obvious, and soil Cd pollution may worsen under the default scenario. Under the pessimistic scenario, the distribution zones for the first and secondary standards of soil Cd pollution gradually decreased and were mainly distributed in areas east of Chengdu. In addition, from now into the future, the distribution areas that meet the national standards for soil Cd pollution will change from expanding to shrinking under the optimistic and default scenarios but will gradually shrink under the pessimistic scenario. These results indicate that the soil Cd pollution in the study zone will worsen and then improve over time under the optimistic and default scenarios, while continual worsening will occur under the pessimistic scenario, especially in Sichuan Province. In both 2050 and 2070, the soil Cd pollution in the study area showed different distributions assuming the three scenario predictions, with the optimistic scenario showing the best results, followed by the default scenario and pessimistic scenario. Significantly, many counties in Meishan and Chengdu will face a severe situation under both the pessimistic and default scenarios; the regions of first-grade soil Cd pollution will further shrink, and secondary areas will be extremely unstable (**Figure 8**). In Shaanxi and Chongqing, the distribution will be relatively stable from 2050 to 2070, with a weak influence, and most zones will still be affected by soil pollution, leading to a very low dominant position for *L. chuanxiong* cultivation.

**Figure 9:**
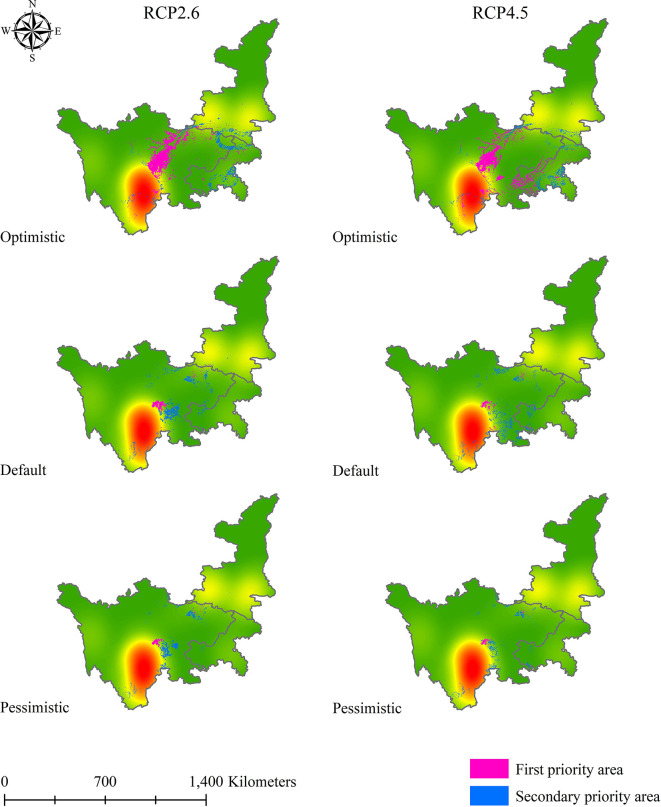
Priority planting area for *L. chuanxiong* under RCP2.6 and RCP4.5 in 2050.

**Figure 10:**
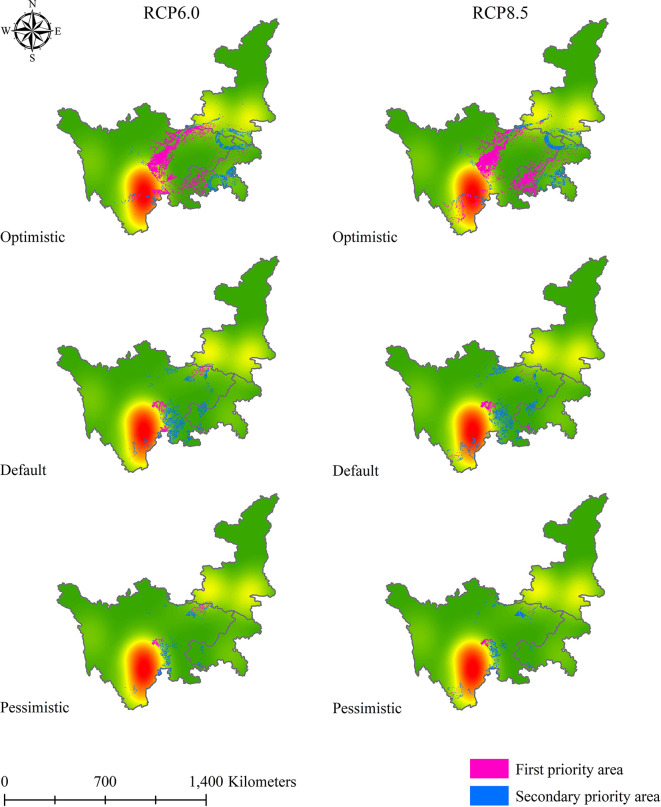
Priority planting area for *L. chuanxiong* under RCP6.0 and RCP8.5 in 2050.

**Figure 11:**
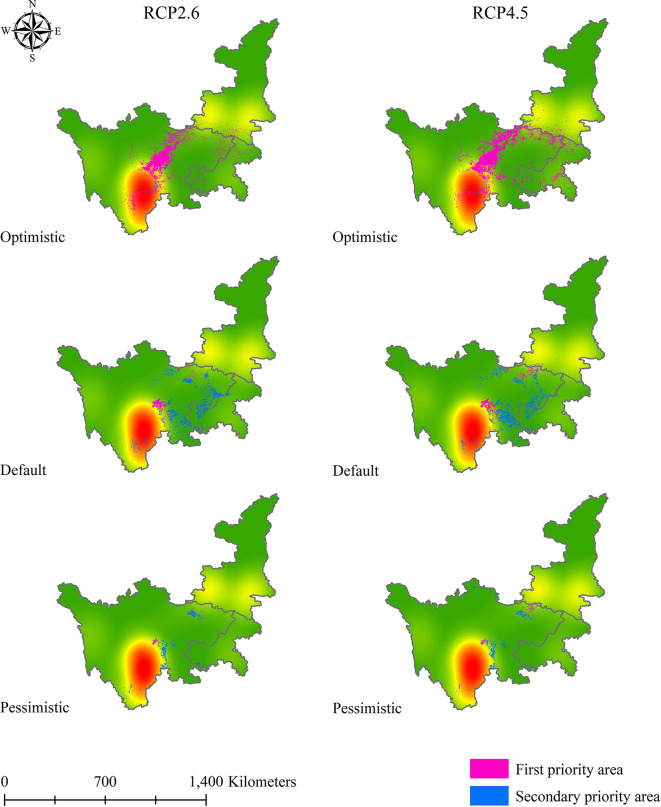
Priority planting area for *L. chuanxiong* under RCP2.6 and RCP4.5 in 2070.

**Figure 12:**
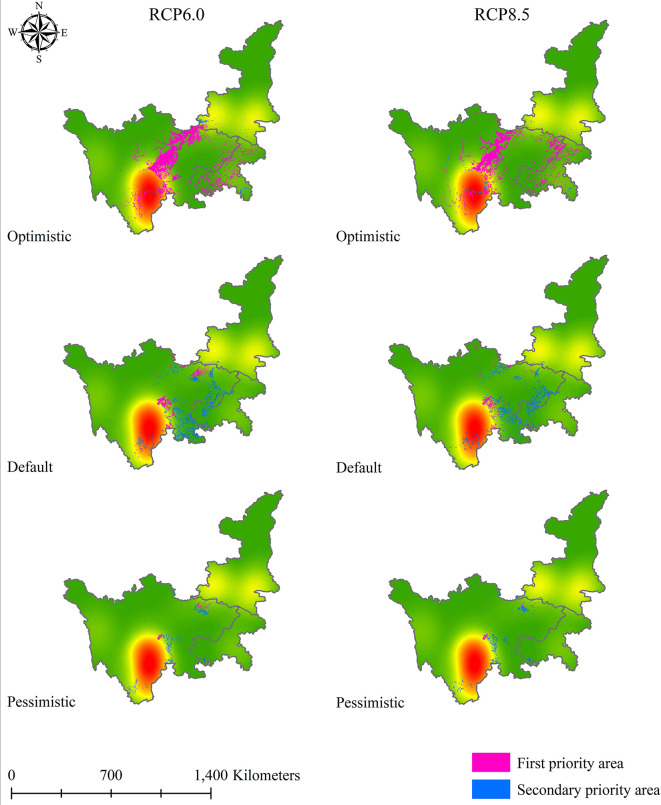
Priority planting area for *L. chuanxiong* under RCP 6.0 and RCP 8.5 in 2070.

After obtaining the results for the first and secondary standards of soil Cd pollution in 2050 and 2070, we applied a similar method to determine the priority suitable zones for *L. chuanxiong* under eight climate change scenarios. **Figures 9**–**12** show that the distribution of priority cultivation areas under the optimistic scenario, including first- and secondary-priority planting zones, is the largest, followed by those under the default scenario and pessimistic scenario. Although the priority planning areas were different, the most stable regions based various climate change and soil Cd pollution scenarios were Chengdu and Meishan, which was highly consistent. Under different time, climate change, and soil Cd pollution scenarios, the first-priority planting regions for *L. chuanxiong* will be centered on Chengdu and Meishan, extending north to the junction of southern Hanzhong and Guangyuan, south to Leshan, west to Barkam and Ya’an, and east to Hechuan, Dazu, Yongchuan, and Jiangjin. For secondary-priority planting zones, the distribution areas showed two main centers, with Meishan, Leshan, Yibin, and Neijiang being particularly important for the future cultivation of *L. chuanxiong*. Guangyuan city will also be the main distribution area in the secondary-priority zone, and some counties, such as Cangxi and Wangcang, will have a relatively stable distribution. In addition, the distribution of secondary-class priority zones for *L. chuanxiong* will be relatively scattered compared with that of the first-class priority areas, and there will be greater uncertainty except in the core regions.

Specifically, the results obtained from statistics for the areas of priority planting regions for *L. chuanxiong* revealed the substantial dominance of different counties. We selected the top ten counties in accordance with the priority areas of *L. chuanxiong* for each climate change and soil pollution scenario to conduct a comparative analysis. Then, the number of occurrences of every county under different soil Cd pollution levels in 2050 or 2070 was counted, and the counties with more than four occurrences among the eight different scenarios were identified (**Table S1**). For instance, under the optimistic scenario of 2050, six counties, Pingwu, Chongzhou, Guangyuan, Shizhu, Pengshui, and Wulong, were identified with four occurrences, leading them to have the same priority in the future site selection of *L. chuanxiong*. For the default scenario of 2050, Renshou County showed eight, the highest number of occurrences, and other counties, such as Meishan, Qionglai, Pujiang, Pengshan, Shuangliu, Jiange, Rong, and Pingwu, were also very suitable for *L. chuanxiong* cultivation. In the pessimistic scenario of 2050, only five counties were identified, with Guangyuan showing seven occurrences, followed by Cangxi, Renshou, and other regions. For 2070, a total of sixteen counties received relative priority during this evaluation under the optimistic scenario of soil Cd pollution. Ziyang and Chongqing are regarded as the most suitable regions for cultivation if the soil Cd pollution in the future is in the default state, followed by Zizhong and Changshou Counties. In the pessimistic scenario of 2070, Yanyuan County appears frequently under various climate change scenarios, and Yanbian and Wangcang Counties will play an important role in the future. As such, different measures of planning, management, and conservation will need to be taken according to the various results reported here, which will be conducive to resource conservation and cash crop improvement.

## 4 Discussion

### 4.1 Ecological characteristics and selection of priority planting areas for *L. chuanxiong*

*L. chuanxiong* is an important cash crop that, according to previous studies (Kim et al., 2020), depends largely on temperature and precipitation; however, different results were obtained in this study. We found that the distribution and growth of *L. chuanxiong* were closely associated with solar radiation, topsoil cec-clay, annual temperature range, altitude, and wind, all of which influenced plant growth. By receiving solar energy in regular daily and seasonal pulses, the earth stores a large amount of energy, while natural ecosystems are the most efficient energy dissipaters (Tsoutsos et al., 2005). Biocenosis and individual species in natural ecosystems continuously convert this absorbed energy *via* succession and evolution, resulting in continuous survival and reproduction (Coscieme et al., 2011; Bassam, 2013; Kiryushin, 2018). Through photosynthesis, solar radiation can be accumulated by living organisms and in biomass, and through the transpiration of water, energy can also be converted into latent heat (Monteith, 1972; Ruimy et al., 1995; Cao, 2018). However, the cation exchange capacity of the surface clay is a critical parameter to consider, as it reflects the cations that can be exchanged with each other in the soil (Chapman, 1965; Khaledian et al., 2017). Previous studies have revealed that sites with deep, neutral sandy soil and rich organic matter are ideal cultivation sites for *L. chuanxiong*, which is consistent with our findings (Kim et al., 2020). Temperature, altitude, and wind also affect the formation of species distribution patterns and affect the quality or production of crops to a certain extent.

Driven by these environmental parameters, the priority planting areas were obtained and combined with occurrence point data, soil Cd pollution, and other factors. In general, the priority planting areas identified using this method were particularly useful because the time period covered by the underlying data was lengthy and did not rely on anthropogenic disturbance. Seven first-priority cultivation counties under the current conditions selected in our study of *L. chuanxiong* (namely, Meishan, Qionglai, Pujiang, Pengshan, Xinjin, Renshou, and Dayi) will be scientifically beneficial for Chinese medicine enterprises and local economic development based on current climate conditions. Our results indicate that if *L. chuanxiong* is grown in these regions, not only will high yields be obtained, but there will also be no threat of soil Cd pollution. Twelve counties, including Pingwu, Hechuan, Jiange, and Fushun, will further improve the production of *L. chuanxiong*; there is great value in expanding the cultivation range and scale of the species. Our findings were consistent with those of previous studies, especially regarding the first-priority planting zones, and they were consistent with the traditional primary production areas under cultivation for thousands of years (Ni et al., 2021; He et al., 2022). In terms of secondary areas, southeastern Sichuan and northwestern Chongqing have greater advantages because of the concentrated distribution of planting areas. Reasonable management should be employed to achieve the desired effect when cultivating *L. chuanxiong*.

### 4.2 Effects of time on priority planting area

In the future, soil Cd pollution will exhibit various changes owing to natural factors and human interference (Li N. et al., 2020; Wang et al., 2020), while the location of the minerals will remain stable over time. Therefore, the priority areas planned for *L. chuanxiong* planting will continuously change in response to the threat of soil Cd pollution and climate change. In general, the results of the default scenario will be more like those in the current situation, while the results of the pessimistic scenario suggest more pressure for *L. chuanxiong* in 2050 and 2070. If a series of energy-saving and emission reduction measures are taken, the future situation of soil Cd pollution will be further improved (Zhang et al., 2015; Rizwan et al., 2016; Zhao et al., 2018). Our results suggested that the suitable habitat of *L. chuanxiong* will also change greatly in the future, and the greatest changes are to the highly suitable and moderately suitable areas. The centroid migration trend in the highly and moderately suitable areas represents the habitat change of *L. chuanxiong*, which is caused by different climate change scenarios (**Figure S4**). As such, we considered these comprehensive factors to identify multiple priority regions for cultivating *L. chuanxiong* under different soil Cd pollution levels in the future; these counties will have high consistency with current counties, including Renshou, Meishan, Qionglai, and other regions (Ren et al., 2015; Peng et al., 2020). Additionally, some counties, including Zhong, Xiaojin, and Yanbian, which are not currently dominant areas for *L. chuanxiong* planting, were identified in this evaluation (Chen et al., 2011). The habitats of these regions should be conserved due to their potential priority and should be considered in future site selection for *L. chuanxiong* planting. To achieve efficient conservation, receive significant financial rewards, and avoid investing resources and possible extinction, future planting management of *L. chuanxiong* should rely more on the results of future planning to make more reasonable decisions.

### 4.3 Planning and management recommendations

Overall, the results indicated that current priority cultivation areas will change greatly owing to the influence of soil Cd pollution and climate change, especially under the pessimistic scenario and RCP2.6. Regarding priority planting, the traditional areas for *L. chuanxiong* planned in this study, including Renshou, Meishan, Qionglai, and other regions, should be protected by adopting conservation methods, such as *in situ* conservation, ex situ conservation, and germplasm conservation (Volis & Blecher, 2010; Braverman, 2014). Dujiangyan and other traditional planting regions beyond the scope of our assessment should be restored by adopting a series of control measures, including physical control, chemical control, and biological control (Posthumus et al., 2015; Seutloali & Beckedahl, 2015; Molla & Sisheber, 2017). For the counties that are not currently the main cultivation areas of *L. chuanxiong* but were identified in this study, appropriate introduction and cultivation should be conducted by decision-makers. The government should formulate relevant policies and cooperate with businesses to encourage farmers to expand planting zones, and at the same time, new crop varieties and planting technologies should be introduced to conduct large-scale cultivation in a scientific and standardized manner (Tester & Langridge, 2010; Zhang X. et al., 2016; Zeng et al., 2020).

In addition, the first seven and twelve secondary-priority counties under current conditions and the number of counties selected in 2050 and 2070 should be recognized as critical areas to develop. However, more factors, such as solar radiation, topsoil cec-clay, annual temperature range, altitude, and wind, should be considered to achieve more efficient protection. Modern agriculture should incorporate the daily demand for growth to carry out corresponding manual intervention according to the range of suitable thresholds of critical environmental parameters (Ferdushi et al., 2013; Cho et al., 2020). For example, the average daily solar radiation should not exceed 12028.92 kJ·m^-2^, the annual temperature range should be between 25.98 and 29.23 °C, and the most suitable range for wind is 1.09 to 1.55 m/s. If these parameters are incorporated into the planting process of *L. chuanxiong*, the yield will be maximized.

### 4.4 Important implications and future outlook

Considering the issues of soil pollution by heavy metals and climate change, understanding the state of species distribution and heavy metal pollution is fundamental in planting and conservation, yet different cash crops have different spatial distributions and are exposed to different types and degrees of soil pollution by heavy metals (Ciarkowska, 2018; Vareda et al., 2019). Consequently, we developed a new synthetic evaluation system with *L. chuanxiong* and soil Cd pollution as an example to plan priority suitable cultivation zones. Our findings are informative for the conservation of *L. chuanxiong*, as well as the current and future management of *L. chuanxiong*. This study was performed in a context without strong human interference and interspecies interactions, which can lead to bias in the results (Haegeman and Loreau, 2011; Yackulic et al., 2015; Martínez-Blancas et al., 2022). We simulated the current and future potential spatial distributions of *L. chuanxiong* in Sichuan, Shaanxi, and Chongqing using the MaxEnt model. Similarly, we simulated the spatial distribution of soil Cd pollution and the influence of minerals through geographic information technology. By coupling these findings with the MaxEnt model results, soil Cd pollution, and the effects of minerals, we created priority planting maps for *L. chuanxiong*. Additionally, in our study, we explored soil Cd pollution in 2050 and 2070 using three scenario prediction models of soil Cd pollution. Then, we utilized the same planning method to determine the priority planting zones in 2050 and 2070 based on different climate change scenarios. Finally, cultivation planning strategies were proposed based on the results of the determined priority suitable zones, ecological characteristics, and spatial dynamic changes, and these strategies will help improve the ecological and economic value of the crop if implemented properly.

Hence, it is necessary to plan and manage planting areas based on a diversified approach under soil pollution by heavy metals and climate change (Bytnerowicz et al., 2007; Sverdrup et al., 2007; Badrzadeh et al., 2022). The sources of soil pollution by heavy metals are diverse, and the soil remediation process is long; this affects the distribution of cash crops and further affect agricultural income and the planting of crops (Dong et al., 2001; Dach and Starmans, 2005; Nabulo et al., 2010; Lu, 2019). We must obtain priority areas for cash crops to meet market demands while ensuring the health and safety of food, while formulating environmental conservation planning strategies (Chen et al., 2014; Qin et al., 2021). The distribution suitability of cash crops is the basis for selecting priority cultivation areas, and then the risk of heavy metal pollution in this region must be evaluated to identify safe areas for use, which will facilitate the reasonable allocation of resources; the restoration of remaining contaminated land should proceed quickly (Alloway, 2001; Delang, 2017; Munishi et al., 2021). Our integrated evaluation system, established based on a spatial niche model, scenario simulation model, and geographic information system, will provide a reference for the planting of all contamination-threatened cash crops. Obtaining an in-depth understanding of the impact of climate change on cash crops and revealing the dynamic process of soil pollution by heavy metals may become major research goals in the future. Thus, planning planting areas for cash crops may require experts in multiple fields, which will greatly contribute to the conservation and management of crops (Munishi et al., 2021). This study had some limitations, including the selection of environmental parameters and niche models, the precise prediction of future soil pollution by heavy metals, and the succession of vegetation caused by climate change. In addition, the soil pollution by heavy metals affecting different cash crops, such as rice and potatoes, may differ (Wang et al., 2003; Cheraghi et al., 2013; Tumanyan et al., 2019). The dynamic change and weight analysis of soil pollution by heavy metals are also difficult problems to address. Our future work will focus on overcoming these technical challenges to obtain more accurate planning results.

## 5 Conclusions

In this study, we successfully established a comprehensive evaluation system for cash crops with *L. chuanxiong* and soil Cd pollution as examples, and priority planting areas under current and future conditions were identified. We developed this evaluation system from an ecological and geographic perspective, using plant and soil pollution by heavy metals as an example. Ultimately, our results indicated that the seven first and twelve secondary counties for current *L. chuanxiong* cultivation should be given higher priority. The spatial dynamics of priority areas in 2050 and 2070 clearly fluctuated based on different prediction scenarios, and critical environmental parameters, such as solar radiation and the annual temperature range, should be considered when planning cultivation. We hope that this evaluation system can be applied to other crops threatened by soil pollution by heavy metals, with the aims of increasing yield and ensuring food safety. Throughout the process of establishing an evaluation system, from the preparation of basic data to the establishment of the model and the interpretation of the results, planning and planting suggestions for economic crops were ultimately proposed. All results can be utilized in the selection and planning management of priority planting areas under the process of current and future cultivation.

## Data availability statement

The original contributions presented in the study are included in the article/**Supplementary Materials**. Further inquiries can be directed to the corresponding author.

## Author contributions

Author contribution statement: PH: Conceptualization, Writing-original draft, Writing – review and editing. YL: Validation. TH, CP and MB provide extensive useful comments. FM provides fund support. All authors contributed to the article and approved the submitted version.
