# Supplementary figures and images for: Priority planting area planning for cash crops under heavy metal pollution and climate change: A case study of Ligusticum chuanxiong Hort
Source: Front Plant Sci. 2023 Feb 1;14:1080881. doi: 10.3389/fpls.2023.1080881 (PMC9928953; doi:10.3389/fpls.2023.1080881)

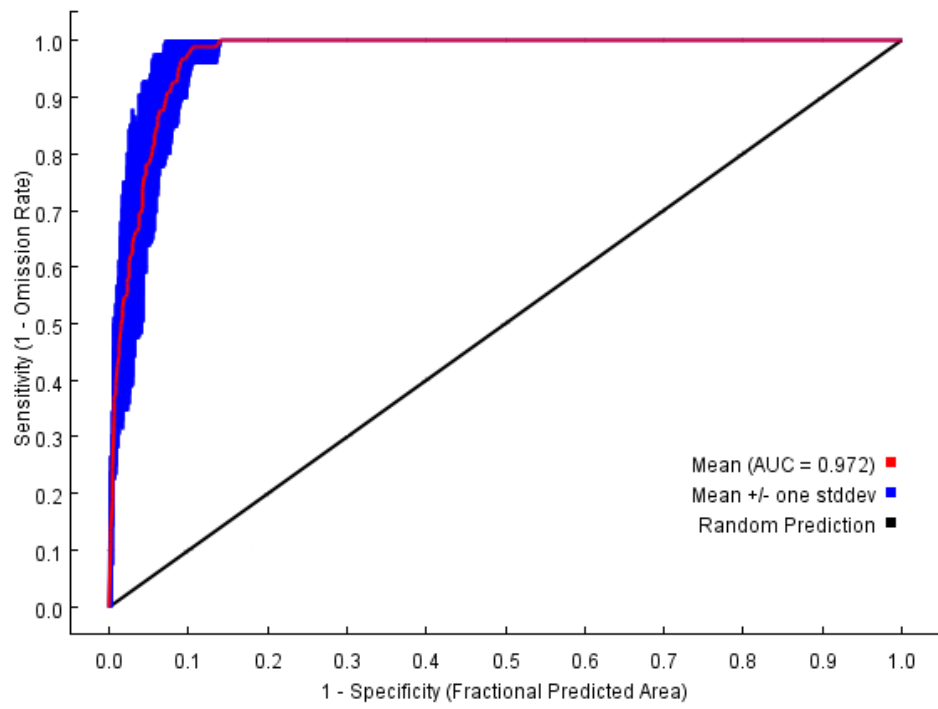

Figure. S1. AUC training values for *L. chuanxiong* under current climate changes.

Supplement: Supplementary file 1 [file Image_1.pdf]

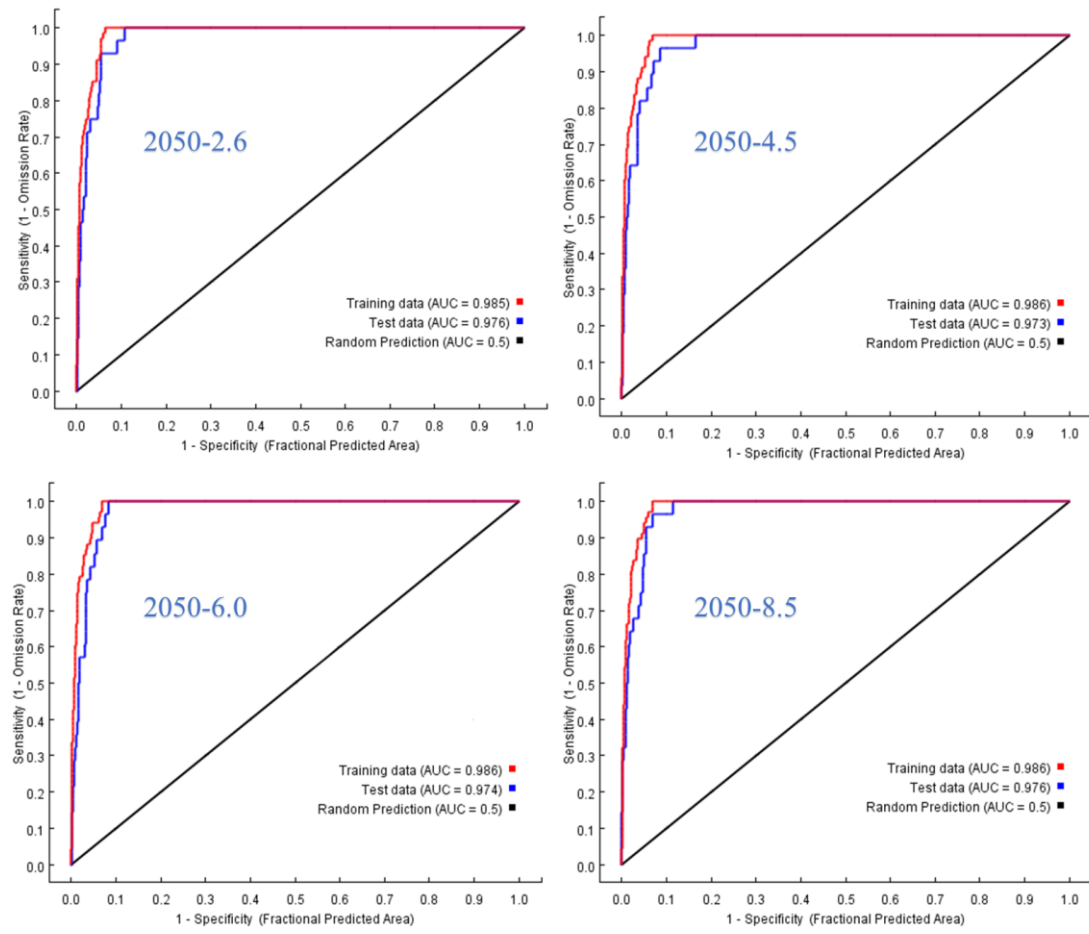

Figure. S2. AUC training values for *L. chuanxiong* in 2050.

Supplement: Supplementary file 2 [file Image_2.pdf]

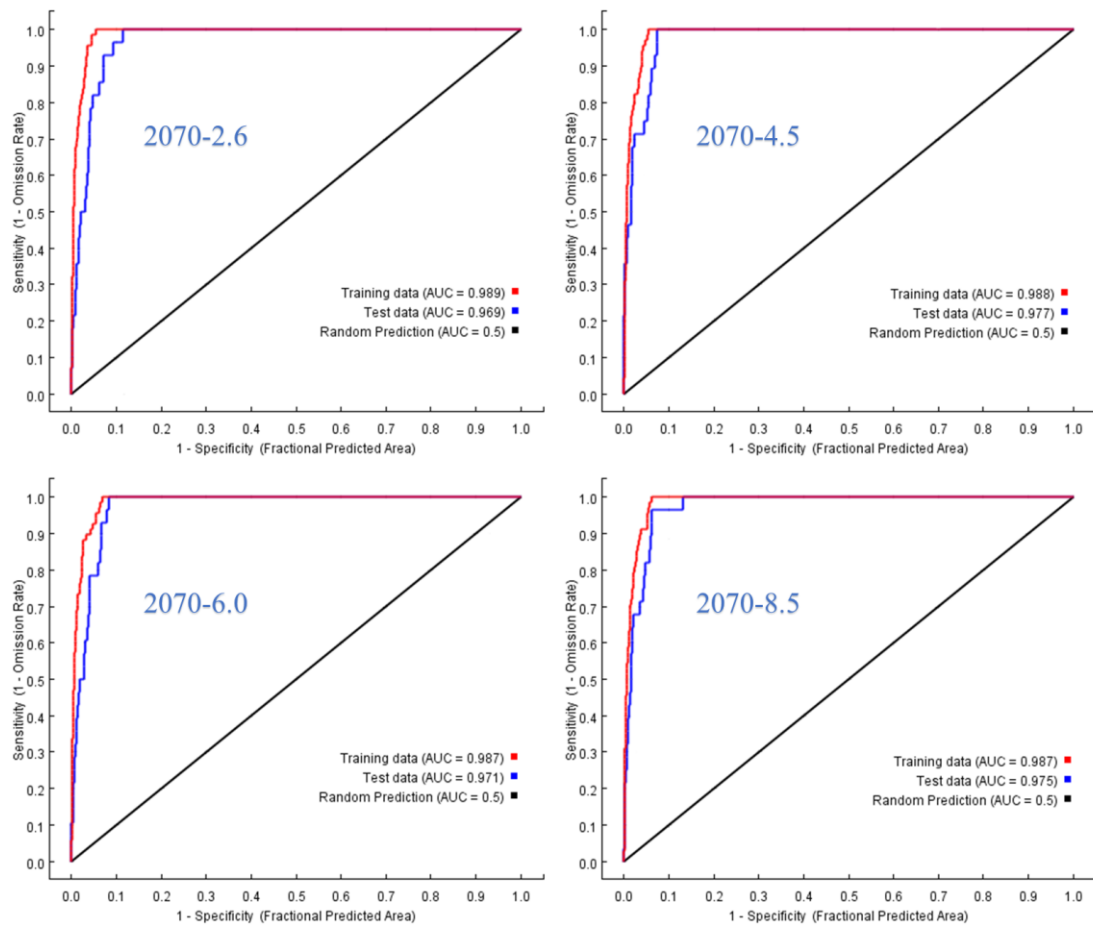

Figure. S3. AUC training values for *L. chuanxiong* in 2070.

Supplement: Supplementary file 3 [file Image_3.pdf]
