# Supplementary material for: Priority planting area planning for cash crops under heavy metal pollution and climate change: A case study of Ligusticum chuanxiong Hort
Source: Front Plant Sci. 2023 Feb 1;14:1080881. doi: 10.3389/fpls.2023.1080881 (PMC9928953; doi:10.3389/fpls.2023.1080881)
Supplement: Supplementary file 4 [file Image_4.pdf]

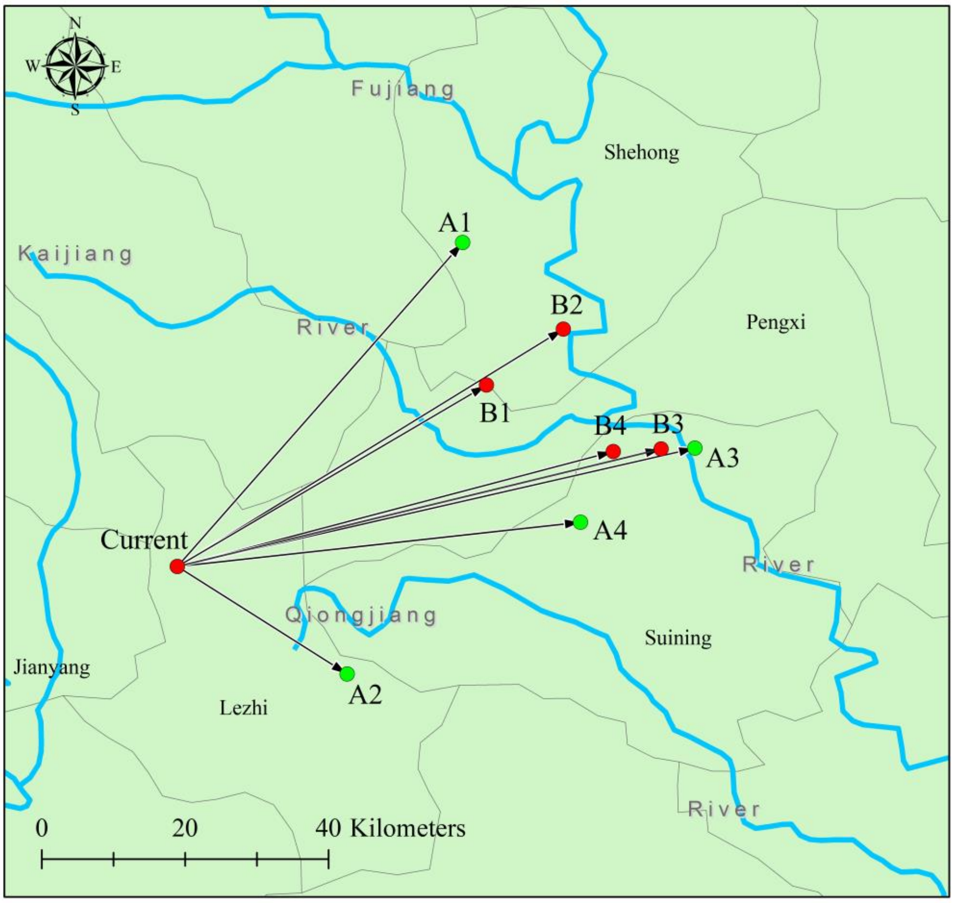

Figure. S4. Migration trend of the centroid of highly and moderately suitable areas for *L. Chuanxiong* under future climate change.
