# Supplementary material for: Priority planting area planning for cash crops under heavy metal pollution and climate change: A case study of Ligusticum chuanxiong Hort
Source: Front Plant Sci. 2023 Feb 1;14:1080881. doi: 10.3389/fpls.2023.1080881 (PMC9928953; doi:10.3389/fpls.2023.1080881)
Supplement: Supplementary file 5 [file Table_1.pdf]

Table S1. Number of occurrences of the top ten counties under each soil cadmium pollution forecast scenario.

County	Occurrences in 2050			Occurrences in 2070		
	Optimistic	Default	Pessimistic	Optimistic	Default	Pessimistic
ZiYang	/	/	/	4	8	4
Chongqing	/	/	/	4	8	4
Pingwu	4	4	/	/	/	/
Guanyuan	4	/	7	/	/	/
Renshou	/	8	4	/	/	/
Jiange	/	4	4	/	/	/
Qionglai	/	4	/	/	/	4
Pujiang	/	4	/	/		4
Wangcang	/	/	4	/	/	6
Zigong	/	/	/	4	4	/
Zhongjiang	/	/	/	4	4	/
Yanyuan	/	/	/	/	4	8
Yanbian	/	/	/	/	4	7
Yibin	/	/	/	/	8	4
Chongqing	4	/	/	/	/	/
Shizhu	4	/	/	/	/	/
Pengshui	4	/	/	/	/	/
Wulong	4	/	/	/	/	/
Pengshan	/	4	/	/	/	/
Meishan	/	4	/	/	/	/
Shuangliu	/	4	/	/	/	/

Rong	/	4	/	/	/	/
Cangxi	/	/	5	/	/	/
Zhong	/	/	/	4	/	/
Zhenping	/	/	/	4	/	/
Zhen’an	/	/	/	4	/	/
Youyang	/	/	/	4	/	/
Xiushan	/	/	/	4	/	/
Xiaojin	/	/	/	4	/	/
Zitong	/	/	/	/	4	/
Zizhong	/	/	/	/	7	/
Changshou	/	/	/	/	7	/
Chnagning	/	/	/	/	4	/
Ningqiang	/	/	/	/	/	4
Nanzheng	/	/	/	/	/	4
Nanping	/	/	/	/	/	4
Nanjiang	/	/	/	/	/	4
Mingshan	/	/	/	/	/	4
Yilong	/	/	/	/	/	4
Xinjin	/	/	/	/	/	4
