# Supplementary material for: Priority planting area planning for cash crops under heavy metal pollution and climate change: A case study of Ligusticum chuanxiong Hort
Source: Front Plant Sci. 2023 Feb 1;14:1080881. doi: 10.3389/fpls.2023.1080881 (PMC9928953; doi:10.3389/fpls.2023.1080881)
Supplement: Supplementary file 6 [file Table_2.pdf]

Table S2. The first priority areas under current climate conditions.

County	Area (km^2)	Proportion (%)
Meishan	577.26	27.98
Qionglai	329.63	15.98
Pujiang	254.8	12.35
Pengshan	180.68	8.76
Xinjin	154.68	7.5
Renshou	135.1	6.55
Dayi	107.33	5.2
Mingshan	62.65	3.04
Shuangliu	58.49	2.83
Guangyuan	48.65	2.36
Ziyang	25.39	1.23
Wangcang	18.2	0.88
Cangxi	15.24	0.74
Qingshen	13.69	0.66
Yanyuan	9.44	0.46
Qingchuan	8.71	0.42
Lingshui	7.86	0.38
Jiangbei	7.67	0.37
Nanping	7.45	0.36
Hechuan	5.72	0.28
Dianjiang	5.49	0.27
Muchuan	4.39	0.21
Nanzheng	4.3	0.21
Jingyan	2.69	0.13
Pingshan	2.61	0.13
Chongqing	2.12	0.1

Yibin	2.08	0.1
Jiange	1.6	0.08
Yanbian	1.5	0.07
Nanjiang	1.45	0.07
Jiangjin	1.24	0.06
Derong	1.05	0.05
Heishui	1.03	0.05
Jianwei	1.01	0.05
Bazhong	0.98	0.05
Miyi	0.65	0.03
Jianyang	0.45	0.02
Hejiang	0.14	0.01
