# Supplementary material for: Priority planting area planning for cash crops under heavy metal pollution and climate change: A case study of Ligusticum chuanxiong Hort
Source: Front Plant Sci. 2023 Feb 1;14:1080881. doi: 10.3389/fpls.2023.1080881 (PMC9928953; doi:10.3389/fpls.2023.1080881)
Supplement: Supplementary file 7 [file Table_3.pdf]

Table S3. The secondary priority areas under current climate conditions.

County	Area (km^2)	Proportion (%)
Pingwu	1112.72	6.8
Lu	979.63	5.98
Hechuan	880.91	5.38
Jiange	856.75	5.23
Longchang	686.28	4.19
Fushun	530.3	3.24
Weiyuan	487.68	2.98
Jiangbei	481.65	2.94
Zizhong	455.98	2.79
Ziyang	447.77	2.73
Renshou	439.53	2.68
Yuechi	404.33	2.47
Linshui	373.54	2.28
Dazhu	371.32	2.27
Rong	352.42	2.15
Guangan	346.29	2.12
Qingchuan	331	2.02
Bishan	318.57	1.95
Shuangliu	285.48	1.74
Tongliang	270.56	1.65
Wusheng	245.4	1.5
Jianyang	237.8	1.45
Da	225.29	1.38
Rongchang	200.2	1.22
Hejiang	189.69	1.16
Li	187.77	1.15

Nanjiang	184.78	1.13
Heishui	184.18	1.12
Neijiang	182.26	1.11
Jiangjin	178.66	1.09
Qu	176.32	1.08
Huaying	175.37	1.07
Ba	156	0.95
Chengdu	148.41	0.91
Nanxi	137.98	0.84
Zigong	127.48	0.78
Cangxi	125.11	0.76
Dayi	118.95	0.73
Anyue	118.62	0.72
Beichuan	113.24	0.69
Lezhi	112.99	0.69
Leshan	109.91	0.67
Mao	105.71	0.65
Chonngqing	102.78	0.63
Yongchuan	93.99	0.57
Qingshen	92.4	0.56
Songpan	92.08	0.56
Qijiang	90.49	0.55
Gao	81.45	0.5
Qionglai	78.82	0.48
Nanzheng	76.95	0.47
Guangyuan	75.53	0.46
Bazhong	70.95	0.43
Kai	69.75	0.43
Yibin	68.84	0.42

Yibin	65.5	0.4
Dianjiang	64.93	0.4
Xuyong	62.05	0.38
Yingshan	59.48	0.36
Liangping	56.86	0.35
Junlian	51.49	0.31
Yilong	50.37	0.31
Gulin	48.67	0.3
Xingwen	47.71	0.29
Pingchang	44.84	0.27
Ningqiang	39.69	0.24
Chongqing	39.23	0.24
Pengan	39.2	0.24
Miyi	38.85	0.24
Naxi	38.45	0.23
Langzhong	36.84	0.23
Pingshan	36.05	0.22
Changshou	34.1	0.21
Kaijiang	32.96	0.2
Changning	29.91	0.18
Gong	25.44	0.16
Zitong	25.02	0.15
Jiajiang	22.9	0.14
Jingyan	21.29	0.13
Jintang	21.26	0.13
Wanxian	20.79	0.13
Meishan	20.62	0.13
Nanbu	20.45	0.12
Jianwei	18.1	0.11

Jiangan	16.97	0.1
Yanyuan	15.88	0.1
Chenggu	15.5	0.09
Pujiang	13.35	0.08
Mingshan	12.52	0.08
Xide	9.99	0.06
Luzhou	8.85	0.05
Nanping	3.37	0.02
Wangcang	2.78	0.02
Suining	2.28	0.01
Nanchong	1.92	0.01
Muchuan	1.6	0.01
Zhongjiang	1.51	0.01
Shiquan	0.96	0.01
Xinjin	0.64	0.004
Santai	0.5	0.003
Ningshan	0.48	0.003
Taibai	0.47	0.003
Pengxi	0.36	0.002
Xixiang	0.22	0.001
Yanbian	0.08	0.0005
Yang	0.02	0.0001
